# FcγRIIIa receptor interacts with androgen receptor and PIP5K1α to promote growth and metastasis of prostate cancer

**DOI:** 10.1002/1878-0261.13166

**Published:** 2022-01-23

**Authors:** Per Flodbring Larsson, Richard Karlsson, Martuza Sarwar, Regina Miftakhova, Tianyan Wang, Azharuddin Sajid Syed Khaja, Julius Semenas, Sa Chen, Andreas Hedblom, Amjad Ali, Kristina Ekström‐Holka, Athanasios Simoulis, Anjani Kumar, Anette Gjörloff Wingren, Brian Robinson, Sun Nyunt Wai, Nigel P. Mongan, David M. Heery, Daniel Öhlund, Thomas Grundström, Niels Ødum, Jenny L. Persson

**Affiliations:** ^1^ Department of Molecular Biology Umeå University Sweden; ^2^ Division of Experimental Cancer Research Department of Translational Medicine Clinical Research Centre Lund University Malmö Sweden; ^3^ Center for Molecular Pathology Lund University Malmö Sweden; ^4^ Department of Clinical Pathology and Cytology Skåne University Hospital Malmö Sweden; ^5^ Department of Biomedical Sciences Malmö University Malmö Sweden Sweden; ^6^ Department of Pathology Weill Cornell Medical College New York NY USA; ^7^ Umeå Centre for Microbial Research (UCMR) Umeå University Sweden; ^8^ Faculty of Medicine and Health Sciences School of Veterinary Medicine and Sciences University of Nottingham UK; ^9^ School of Pharmacy University of Nottingham UK; ^10^ Wallenberg Centre for Molecular Medicine and Department of Radiation Sciences Umeå University Sweden; ^11^ Department of Immunology and Microbiology University of Copenhagen Denmark

**Keywords:** AR pathway and antibody‐based therapy, FcγRIIIa receptor, PIP5K1α, prostate cancer metastasis, targeted therapy

## Abstract

Low‐affinity immunoglobulin gamma Fc region receptor III‐A (FcγRIIIa) is a cell surface protein that belongs to a family of Fc receptors that facilitate the protective function of the immune system against pathogens. However, the role of FcγRIIIa in prostate cancer (PCa) progression remained unknown. In this study, we found that FcγRIIIa expression was present in PCa cells and its level was significantly higher in metastatic lesions than in primary tumors from the PCa cohort (*P* = 0.006). PCa patients with an elevated level of FcγRIIIa expression had poorer biochemical recurrence (BCR)‐free survival compared with those with lower FcγRIIIa expression, suggesting that FcγRIIIa is of clinical importance in PCa. We demonstrated that overexpression of FcγRIIIa increased the proliferative ability of PCa cell line C4‐2 cells, which was accompanied by the upregulation of androgen receptor (AR) and phosphatidylinositol‐4‐phosphate 5‐kinase alpha (PIP5Kα), which are the key players in controlling PCa progression. Conversely, targeted inhibition of FcγRIIIa via siRNA‐mediated knockdown or using its inhibitory antibody suppressed growth of xenograft PC‐3 and PC‐3M prostate tumors and reduced distant metastasis in xenograft mouse models. We further showed that elevated expression of AR enhanced FcγRIIIa expression, whereas inhibition of AR activity using enzalutamide led to a significant downregulation of FcγRIIIa protein expression. Similarly, inhibition of PIP5K1α decreased FcγRIIIa expression in PCa cells. FcγRIIIa physically interacted with PIP5K1α and AR via formation of protein–protein complexes, suggesting that FcγRIIIa is functionally associated with AR and PIP5K1α in PCa cells. Our study identified FcγRIIIa as an important factor in promoting PCa growth and invasion. Further, the elevated activation of FcγRIII and AR and PIP5K1α pathways may cooperatively promote PCa growth and invasion. Thus, FcγRIIIa may serve as a potential new target for improved treatment of metastatic and castration‐resistant PCa.

## Graphical abstract

Our findings show that FcγRIIIa promotes prostate cancer growth and invasion through its direct interaction with androgen receptor. There is also an interaction between FcγRIIIa in cancer cells and IgG immune complexes from the tumor‐associated microenvironment. The increased expression of *FcγRIIIa* may be required for the activation of the PIP5K1α/PI3K/AKT pathways in cancer cells during the progression from androgen‐dependent cancer to a castration‐resistant state.

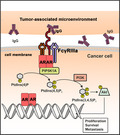

**Abbreviations**
ALDH: aldehyde dehydrogenases
AR: androgen receptor
ARE‐Luc vector: androgen‐responsive element luciferase vector
BPH: benign prostate hyperplasia
BRFS: biochemical recurrence‐free survival
CRPC: castration‐resistant prostate cancer
DHT: dihydrotestosterone
FcγRIIIa: low‐affinity immunoglobulin gamma Fc region receptor III‐A
GDPR: The General Data Protection Regulation
PCa: prostate cancer
PIP2: phosphatidylinositol‐4,5‐P_2_
PIP5K1α: phosphatidylinositol‐4‐phosphate 5‐kinase type‐1 alpha

## 1 Introduction

Fc receptors are a family of cell surface receptors that are commonly expressed by the cells in the immune system against pathogens [1, 2]. FcγRIIIa (CD16a) is an activating Fc receptor and is mainly expressed by mast cells, macrophages, neutrophils, and NK cells [3, 4]. The activation of FcγRIIIa is in part dependent on its binding to the Fc portion of IgG1 antibody as shown in cocrystal structure of FcγRIIIa in complex with IgG [1].

The increased FcγRIIIa expression in monocytes/macrophages is associated with the increased cytokine production that may trigger the inflammatory and autoimmune disease conditions [5, 6, 7, 8, 9, 10, 11]. The spontaneously expansion of the circulating monocytes expressing FcγRIIIa was detected in patients with metastatic gastrointestinal carcinoma [12]. The expanded monocytes expressing FcγRIIIa have also been found in the peripheral circulations of patients with breast cancer [13]. The FcγRIIIa level was increased in blood serum from mice bearing xenograft tumors compared with mice without tumors, suggesting that FcγRIIIa expression may be associated with tumorigenesis [14]. Interestingly, FcγRIII expression was detected in prostate cancer (PCa) cell lines including LNCaP and PC‐3 cells using flow cytometry analysis [15].

Recent advances in tumor immunology suggest that various types of tumors are able to escape from immunological attack by hijacking the key factors from immune cells. It is believed that tumor cells by expressing Fc receptor can block B‐cell normal function, thereby allowing tumor cells to escape T‐cell‐mediated cytotoxicity [16]. However, the expression and localization of FcγRIII in cancer cells and its role in tumorigenesis remains obscure.

Mice with deletion of FcγRIIIa allele had better survivals from the severe sepsis compared with the wild‐type controls [17]. Moreover, mice lacking FcγRIIIa allele had reduced phagocytosis activity and decreased pro‐inflammatory cytokine production in their blood cells in response to E. coli bacteria infection [18]. The monoclonal antibody mAb 3G8 against FcγRIIIa has shown promising effect on autoimmune diseases, as mAb 3G8 is able to induce clearance of the inflammatory immune complexes by selectively blocking FcγRIIIa binding to IgG [19, 20]. A bispecific‐monoclonal antibody (2B1) was produced to against both c‐erbR‐2 onco‐protein and FcγRIII protein, and 2B1 treatment suppressed growth of SK‐OV‐3 human ovarian tumors in xenograft mice without obvious toxicity [21]. These findings provide evidence, supporting a role of FcγRIIIa not only in autoimmune diseases but also in tumorigenesis.

The previous reported studies have suggest that FcγRIIIa may be functionally linked to the lipid kinase phosphatidylinositol‐4‐phosphate 5‐kinase alpha (PIP5K1α) and phosphatidylinositol‐4,5‐P_2_(PIP2) [3, 22, 23, 24]. In the inflammatory cells, FcγRIIIa can induce cytokine production by activating downstream PI3K/AKT pathways [25, 26, 27]. We have previously shown that PIP5K1α is a key regulator that triggers the constitutive activation of PI3K/AKT pathways in PCa cells during tumor growth and invasion [28, 29]. Conversely, the PIP5K1α inhibitor termed ISA‐2011B had significantly inhibitory effect on invasive PCa in xenograft mouse models [28, 30, 31, 32]. However, it remains largely unknown whether PIP5K1α and FcγRIIIa are functionally associated in PCa cells.

In this study, we reported our novel findings on the identification of FcγRIII expression in primary cancer and metastatic tissues from PCa patient cohorts and in various subtypes of PCa cell lines. We demonstrated that FcγRIII is functionally associated with PIP5K1a/AKT and AR pathways and promoted tumor growth and invasion. We further shown that targeted inhibition of FcγRIIIa via siRNA‐mediated knockdown or using inhibitory antibody suppressed growth of primary prostate tumors and reduced distant metastasis in xenograft mouse models. Our findings provide important information on new targets and options for combinational‐targeted therapies for treatment of metastatic PCa.

## 2 Materials and methods

### 2.1 Tissue specimens, tissue microarrays, cDNA microarrays, and CGH arrays

Tissue microarrays (TMAs) containing benign prostatic hyperplasia (BPH) (*n* = 48) vs. matched PCa tissues (*n* = 48) from a patient cohort (*n* = 48 patients), and primary PCa (*n* = 14) and metastatic PCa lesions in different organs including lymph node, liver, lung and bone/bone marrow (*n* = 43) from 14 PCa patients were constructed at Skåne University Hospital, Malmö. The mRNA expression and copy number alteration data were extracted from the two cohorts of MSKCC datasets (*n* = 181 primary; *n* = 37 metastatic prostate cancer samples) [35, 36, 37, 38], the SU2C/PCF metastatic patient cohort (*n* = 429 cases) [33], and the TCGA cohort (*n* = 333 cases) [34] from the Prostate Oncogenenome Project dataset in cBioPortal databases [35, 36, 37, 38]. This study was approved by the Ethics Committee, Lund University and Umeå University. The General Data Protection Regulation (GDPR) was applied, and written informed consent was obtained when required. The Helsinki Declaration of Human Rights was strictly observed.

### 2.2 Immunohistochemical analysis

Immunohistochemistry on tumor tissue microarrays was performed as previously described [39]. The staining procedure was performed using a semiautomatic staining machine (Ventana ES; Ventana Inc.). The staining intensity was scored as 0 (negative), 1 (weakly positive or positive), 2 (moderate positive), or 3 (strongly or very strongly positive). The specimens were evaluated and scored by three different scientists, one of them a specialist in pathology. To evaluate the metastatic invasion of PCa cells in the bone/bone marrow of mice, femurs were fixed in 4% paraformaldehyde for 24 h before decalcification in formic acid and embedded in paraffin sections.

### 2.3 Cell culturing and treatments

PC‐3 (RRID:CVCL_0035), U‐937 (RRID:CVCL_0007), VCaP (RRID:CVCL_2235), LNCaP C4‐2 (RRID:CVCL_4782), and LNCaP (RRID:CVCL_0395) cell lines were purchased from American Type Culture Collection (Manassas, VA, USA). PNT1A (RRID:CVCL_2163) cell line was purchased from Sigma‐Aldrich (Stockholm, Sweden). An androgen‐insensitive cell line, PC‐3M (RRID:CVCL_9555) [40], was kindly provided by Dr. J Fidler (Department of Urology, MD Andersson Cancer Center, TX, USA). All human cell lines were purchased within the last three years or have been authenticated using STR profiling within the last three years. All experiments were performed with mycoplasma‐free cells. For treatment with dihydrotestosterone (DHT), medium containing 10% charcoal‐stripped serum, and 5 nm DHT or vehicle control 0.1% dimethyl sulfoxide (DMSO, Sigma‐Aldrich) were used. Cells were treated with DHT for 12 h. For treatment with enzalutamide (MDV3100) (Selleck Chemicals), cells were treated with enzalutamide at 10 µm for 6, 12, and 24 h or vehicle control 0.1% DMSO. PIP5K1 alpha inhibitor: ISA‐2011B, a diketopiperazine fused C‐1 indol‐3‐yl substituted 1,2,3,4‐tetrahydroisoquinoline derivative at final concentrations of 25 or 50 µm in 0.125% or 1% DMSO was used for treatment for 48 h. For treatment with antibodies, PCa cells or tumor spheroids were treated with purified anti‐human FcγRIIIa monoclonal antibody (M3G8) or IgG1 isotype control at 500 ng·mL^−1^ (BioLegend, USA).

### 2.4 Mouse models of PCa and PCa distant metastasis

The animal studies were approved by the Swedish Regional Ethical Animal Welfare Committee. Three sets of mouse experiments were performed. Athymic NMRI‐*Foxn1^nu^* nude mice were purchased from the Charles River Laboratories (Sulzfeld, Germany). The male mice (*n* = 4–6, per experiment group) aged 4–6 weeks and weight 25‐27 gram each were used in each experimental setting. (a) 1 × 10^6^ PC‐3M cells transfected with scrambled‐siRNA control or FCGR3A‐siRNA were implanted subcutaneously into left flank of each nude mouse. Growth and invasion of tumors in xenograft mice was assessed (*n* = 4 mice/group). (b) For *in vivo* antibody treatment of subcutaneous PCa tumors, 1 × 10^6^ PC‐3 cells were injected subcutaneously into left flank of each mouse (*n* = 4 per group). (c). For antibody treatment of metastatic PCa, 40 tumor spheroids derived from ALDH^high^ PC‐3M cells were injected subcutaneously into left flank of each mouse (*n* = 4 or 6 per group), and two independent experiments were performed. To assess the frequency of PCa cells metastasized to the distant organs, xenograft mice were injected with HLA‐ABC antibody conjugated with 680‐DyLight NHS‐ester (Life Technologies, Stockholm, Sweden) 6 h before imaging. The *in vivo* imaging device (IVIS imaging system, PerkinElmer, USA) was used. For treatment of mice with purified anti‐human FcγRIIIa (M3G8) monoclonal antibody (M3G8 leaf antibody, BioLegend, USA) at 5 mg·kg^−1^ or isotype IgG1 via intraperitoneal injection were used twice a week. For quantitative analysis of tumor size, Living image ^®^ software was used to measure metastatic areas and signal intensities. Bone/bone marrow samples were collected postmortem and used for immunohistochemical and immunoblot analyses. The animal experiments were under the license numbers: A‐12‐16, A‐13‐16, and A3‐19 approved by the Swedish Regional Ethical Committee. The animal welfare and guidelines were strictly followed. All experimental mice were kept in the ventilation cages under highly sterile conditions with 12‐h light/dark cycles. The maximum number of mice was limited to four per cage. The diet and water for feeding the animals were highly sterile.

### 2.5 Plasmids, transfection, and siRNA knockdowns

For siRNA‐mediated knockdown, siRNA‐negative control duplex, on‐target plus nontargeting control pool siRNA, siRNAs single oligoes, or on‐target siRNA SMART pool against FCGR3A (Thermo Fisher Scientific, Waltham, MA, USA and GE Health Dharmacon Inc. Lafayette, CO, USA) were used. SiRNAs (50 nm) were transfected into 1 × 10^5^ PCa cells using Transfection Reagent TransIT‐TKO^®^ according to manufacturers’ protocol (Mirus Bio LLC). After introduction of respective siRNA complexes into PCa cells, cells were then collected after 24–48 h post‐transfection. TransIT‐TKO^®^ was used according to the manufacturer's instructions. For transient transfection study to induce overexpression of FcγRIIIa, PIP5K1α or AR into PCa cells, pLX304‐CD16A, pLPS‐3’EGFP‐PIP5K1α, and pLPS‐3’EGFP (PlasmID, Harvard Medical School, MA, USA) were used. AR plasmid vector: pCMV‐AR and control pCMV vector were kindly provided by Prof. Yvonne Gwercman, Department of Translational Medicine, Lund University. Transient transfection was performed using Lipofectamine^®^ 2000/3000 transfection reagent (Life Technologies, UK), TransIT‐2020 or TransIT‐X2^®^ (Mirus Bio, MIR5410, USA) by following the manufacturer's instructions.

### 2.6 Immunoblot and immunoprecipitation analyses

Immunoblot and immunoprecipitation analyses were performed as described previously [40]. Briefly, protein from different subcellular fractions (cytoplasmic and nuclear) was isolated by using NE‐PER™ Nuclear and Cytoplasmic Extraction Reagents according to manufacturer’s protocol (Thermo Fisher Scientific). Densitometric quantification of immunoblots was performed by using the imagej Image Analysis Software (NIH, Baltimore, USA). For immunoblot analysis, antibodies against FCGR3A (CD16a) were purchased from Biosite. For immunoprecipitation analysis, antibody against PIP5K1α was used to pull down the immune complexes, and antibody to IgG (Thermo Fisher Scientific, Sweden) was used as a negative control.

### 2.7 ALDEFLUOR assay

Aldehyde dehydrogenases (ALDH) expression in PC‐3M cells is used for define the cancer stem cell enriched population ALDH^high^ vs. noncancer stem cell population ALDH^low^ populations. The ALDH^high^ and ALDH^low^ were sorted from PC‐3M cells on FACS Aria (BD Biosciences) as previously described [40]. The ALDEFLUOR Kit (StemCell Technologies, Vancouver, British Columbia, Canada) was used according to manufacturers’ protocol.

### 2.8 Tumor‐spheroid formation assay

5 × 10^3^ PC‐3 cells were prepared in single cell suspensions and were seeded in 2 mL modified medium in 35 mm polyHEMA‐coated culture dishes for 10 to 14 days. The modified medium contains DMEM F‐12, 3.151 g·L^−1^ glucose, L‐glutamine, 1‐2 × B27, 20‐40 ng·mL^−1^ EGF, and 20‐40 ng·mL^−1^ FGFβ.

### 2.9 Immunofluorescence analysis

For staining with primary and secondary antibodies, alternatively, cell suspensions were fixed on slides in methanol in −20 °C for 10 min. PCa cells were grown on glass coverslips in phenol red‐free RPMI‐1640 medium containing 10% FBS for 24 h and fixed with 4% paraformaldehyde in PBS. The slides were stained with primary antibodies. Primary antibodies including anti HLA‐ABC conjugated with FITC and anti‐FCGR3A (CD16a), was purchased from Biosite (Bioss MA, USA), and PIP5K1α (Protein Technologies, UK) was used. Secondary antibodies include anti‐rabbit conjugated to Alexa Fluor 488 (Invitrogen, Stockholm, Sweden), anti‐mouse conjugated to Alexa Fluor 546 (Invitrogen, Stockholm, Sweden), and anti‐rabbit conjugated to Rhodamine (Chemicon International Inc, Temecula, CA). Cells were counterstained with DAPI (4',6‐diamidino‐2‐phenylindole, dihydrochloride) (SERVA Electrophoresis GmbH, Heidelberg, Germany). The cells were examined under an Olympus AX70 microscope using NIS Elements f 2.20 software or a Zeiss Apoptome microscope (Zeiss, Germany) and the zen 2.3 lite software (Zeiss, Germany).

### 2.10 RNA isolation and polymerase chain reaction

RNA was isolated using TRIzol™ reagent (Invitrogen, Carlsbad, CA). For cDNA synthesis, Maxima First Stand cDNA Synthesis Kit was used (Thermo Scientific). The following primers were used as follows: CD16a (NM_001127593.1)—forward: 5’‐GAC AGT GTG ACT CTG AAG‐3’; reverse: 5’‐GCA CCT GTA CTC TCC AC‐3’; GAPDH—forward: 5'‐GGA TTT GGT CGT ATT GGG‐3'; reverse: 5'‐GGA AGA TGG TGA TGG GAT T‐3'. The resultant PCR products were then subjected to electrophoresis and visualized using Proxima C16Phi+ bio‐imaging system (Isogen Life Science). Semiquantifications of the results were performed using ImageJ program.

### 2.11 Proliferation assay

Proliferation of the cells was determined using MTS proliferation assays (Promega Biotech Sweden, Stockholm, Sweden) according to manufacturer’s protocol. Cells at 5 × 10^3^ cells per well were cultured in a 96‐well plates for 48 h. MTS incorporation into the DNA was determined by measuring the absorbance at both 490 nm on an ELISA plate reader Infinite^®^ M200 multimode microplate reader (Tecan Sunrise™, Tecan Group, Männedorf, Switzerland).

### 2.12 Luciferase assays

PC‐3 cells were transiently transfected with different vectors along with the reporter vector containing luciferase gene (Luc) or full‐length cyclin A1 promoter in Luc reporter vector (cyclin A1‐Luc) as indicated. The Firefly Luciferase and Renilla Luciferase activity were determined by using an Infinite^®^ M200 multimode microplate reader (Tecan Sunrise™), equipped with dual injector. For AR receptor activity assay in LNCaP cells, AR Cignal Reporter Assay Kit (Qiagen Inc.) was used according to the manufacturer’s protocol. Briefly, LNCAP cells were transiently transfected with different vectors along with the reporter vector ARE‐luciferase (ARE‐Luc) vector as indicated. The Firefly and Renilla Luciferase with Dual Luciferase Assay Kit (Promega, Biotech Sweden, Stockholm, Sweden) according to standard protocol in the Tecan Infinite M200 (Tecan Trading AG, Mannedorf, Switzerland) plate reader equipped with dual injector.

### 2.13 Migration assay

Cell migration assays were performed using Transparent PET Membrane chambers (Corning, Germany). A total of 0.5–2 × 10^5^ cells in RPMI‐1640 phenol red‐free and serum‐free medium were seeded in the upper chamber, and RPMI media supplemented with 50% serum as a chemoattractant was loaded in the lower chamber to allow the migration to proceed. The migrated cells were stained after 18 h or 24 h, and the proportion of migrated cells was calculated as described [41].

### 2.14 Statistical analysis

Tukey‐test, *t*‐test, Kruskal–Wallis/ANOVA, and Spearman rank correlation tests were performed. Student *t*‐test was used for statistical analyses of the experimental data. The standard deviation (SD) is an indication of variability of all samples. The precision of the sample mean is indicated by standard error. Confidence levels are expressed using 95% confidence interval (CI). All statistical testes were two‐sided, and *P*‐values less than 0.05 were considered to be statistically significant. Data presented are representative of at least two or three independent experiments. Distribution of overall survival (OS) or disease‐free survival/biochemical recurrence‐free survival (BRFS) was estimated by the method of Kaplan–Meier, with 95% confidence intervals. Differences between survival curves were calculated using the log‐rank test. Statistical software, Social Sciences software (spss, version 21, Chicago), and graphpad Software were used.

## 3 Results

### 3.1 Clinical relevance of FcγRIIIa expression in patients with PCa metastasis and its correlation with PIP5K1α

**Fig. 1:**
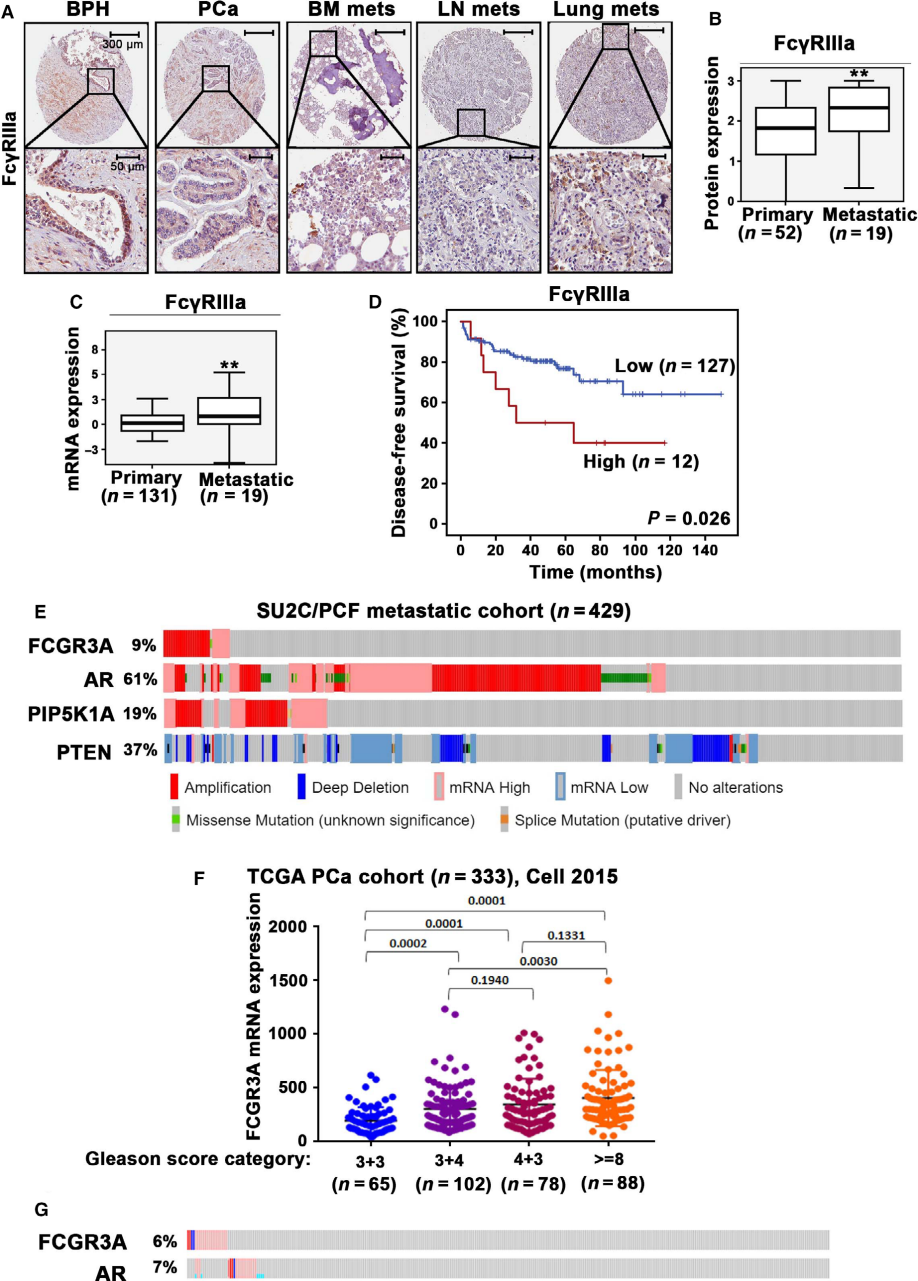
Evaluation of FcγRIIIa expression in primary tumors and metastatic lesions from PCa patients and the association between FcγRIIIa expression and patient outcome. (A) Representative microphotographs of benign prostate hyperplasia (BPH) (*n* = 48), PCa specimens (PCa) (*n* = 52) and metastatic lesions in bone marrow (BM mets), lymph node (LN mets), and lung (Lung mets) from PCa patients (*n* = 19), as assessed by immunohistochemical analysis of TMAs using antibody against FcγRIIIa. The scale bars: 300 µm and 50 µm are indicated and apply to all images in the panel. (B) Box‐plot quantitative comparison of FcγRIIIa protein expression between primary PCa, *n* = 52 and metastatic PCa, *n* = 19 (mean scores in primary and metastatic lesions were 1.73 and 2.17, difference = 0.74; 95% CI = 1.81–2.27, *P* = 0.006). **P < 0.01 is indicated. The error bars indicate SD. The student *t*‐test was used to determine the significance. (C) Box‐plot quantitative comparison of FCGR3A mRNA expression between primary (*n* = 131) and metastatic lesions (*n* = 19) (*P* < 0.01), **P < 0.01 is indicated. The error bars indicate SD. The student *t*‐test was used to determine the significance. (D). Kaplan–Meier survival analysis based on biochemical recurrence‐free (BCR‐free) survival shows the difference between patients with low and high expression of FCGR3A. Differences in BCR‐free survival between two groups and *P‐*values were calculated using the log‐rank test. *P = *0.026 is indicated. (E). Alterations in genes and mRNA expression of FCGR3A, AR and PIP5K1A and PTEN in metastatic PCa cohort SU2C/PCF (*n* = 429) are shown in onco‐prints. Different types of alterations in genes and their respective mRNA expression are indicated. (F). Dot plot graph shows the FCGR3A mRNA expression in four subgroups of PCa that were categorized using Gleason scores (TCGA PCa cohort, *n* = 333). Subgroups with Gleason score 3 + 3 (*n* = 65), Gleason score 3 + 4 (*n* = 102), Gleason score 4 + 3 (*n* = 78), and Gleason score >=8 (*n* = 88). The expression of FCGR3A mRNA in comparison between group of Gleason score >=8 and Gleason score 3 + 4, *P* = 0.003; the expression of FCGR3A in comparison between group of Gleason score 4 + 3 and Gleason score 3 + 3, *P* < 0.001. The ANOVA test was used to determine the significance. (G). Alterations in genes and mRNA expression of FCGR3A and AR in the PCa cohort mentioned in (F) are shown in onco‐prints.

To investigate the role of FcγRIIIa in tumorigenesis and progression of PCa, we firstly examined FcγRIIIa expression in primary tumors and metastatic lesions from PCa patients. FcγRIIIa protein expression was assessed by using immunohistochemical analysis on the tissue microarrays (TMAs) consisting of benign prostate hyperplasia (BPH), adjacent primary PCa and metastatic PCa lesions in lymph nodes, bone marrows, and lungs from patients with primary and metastatic PCa. FcγRIIIa expression was found in the epithelium of BPH tissues (Fig. 1A). Further, FcγRIIIa was expressed by the primary cancer tissues and its expression was nearly significantly higher in the primary PCa tissues as compared with that in the BPH (*P* = 0.051; Fig. 1A). We found that FcγRIIIa was highly expressed in the metastatic lesions in the bone marrows, lymph nodes, and lungs from patients who suffered metastatic PCa (Fig. 1A). Statistical analysis revealed that FcγRIIIa protein expression was significantly higher in metastatic lesions compared with that of primary tumors (*P* = 0.006) (Fig. 1B). We next examined FcγRIIIa mRNA expression in the MSKCC patient cohort from the Prostate Oncogenenome Project dataset in cBioPortal databases [35]. Similar to its protein expression in PCa tissues, FcγRIIIa mRNA expression was found in primary and metastatic PCa from the MSKCC cohort which contained over 95% of cancer cells in the tumor tissues. Further, the expression of FcγRIIIa mRNA was significantly higher in metastatic lesions (*n* = 19) than that of primary tumors (*n* = 131) (*P* < 0.01; Fig. 1C). We have previously reported that PIP5K1α is a key player in PCa progression and metastasis. We therefore wanted to assess whether elevated level of FcγRIIIa expression might be associated with abnormal PIP5K1α expression in PCa primary and metastatic cancer tissues. Spearman’s rank correlation test was performed, which revealed that there was a statistically significant correlation between FcγRIIIa and PIP5K1α protein expression in primary and metastatic cancer tissues from the PCa patient cohorts (*R* ^2^ = 0.480, *P* < 0.001; Table 1). In addition, FcγRIIIa expression correlated with cyclin A1 in cancer tissues from the same patient cohorts (*P* = 0.001; Table 1). To further assess the clinical importance of FcγRIIIa expression, we examined the association between FcγRIIIa expression and biochemical recurrence (BCR)‐free survival of the patients using Kaplan–Meier survival analysis. We found that PCa patients with higher FcγRIIIa mRNA expression in their tumors (*n* = 12) had worse biochemical recurrence (BCR)‐free survival compared to those with lower FcγRIIIa expression (*n* = 127) (*P* = 0.026) (Fig. 1D). Next, we wanted to assess whether alterations in *FCGR3A* gene encodes for FcγRIIIa might be a frequent event in metastatic PCa. To this end, we examined genomic alterations in *FCGR3A* along with *AR, PIP5K1A,* and *PTEN* using a large SU2C/PCF metastatic PCa cohort (*n* = 429) [33]. We found that *FCGR3A* gene amplifications and mRNA upregulation account for 9% of the metastatic PCa cases. Interestingly, 61% cases of this cohort had AR gene amplifications and mRNA upregulation, and 19% had *PIP5K1A* gene amplification and mRNA upregulation. Conversely, *PTEN* gene mutation, deletion, or mRNA downregulation accounted for 37% of this metastatic PCa cohort (Fig. 1E). These data indicate that *FCGR3A* amplification and mRNA upregulation are likely associated with PCa metastatic status. To further assess the clinical importance of FcγRIIIa mRNA expression in PCa progression, we examined FcγRIIIa mRNA expression in primary tumors from the TCGA PCa cohort, which were divided into four subgroups based on the Gleason grade scores (*n* = 333 cases) [34]. FcγRIIIa mRNA expression was significantly higher in the primary tumors with Gleason scores higher than 8 compared with those with lower Gleason grades (3 + 3 or 3 + 4) (*P* < 0.01) (Fig. 1F). These data showed that FcγRIIIa mRNA expression was elevated in the advanced PCa, suggesting the clinical importance of FcγRIIIa expression in PCa. Gene alterations in the *FCGR3A* loci were found in 6% of this PCa cohort, which was similar to what was observed for AR (Fig. 1G). In the MSKCC/DFCI patient cohort consisting primary PCa (*n* = 1013 PCa cases) from the Prostate Oncogenenome Project dataset in cBioPortal databases [42], *FCGR3A* gene alterations were found in 3% of PCa cases, which was similar to *PIP5K1A* gene alterations accounted for 5% in this PCa cohort (Fig. S1). No statistically significant correlations between AR and FcγRIIIa mRNA expression were found in SU2C/PCF metastatic PCa cohort and TCGA cohort (Figs. S2,S3).

**Table 1:** Correlations for protein expression in patient samples.

FcγRIIIa and PIP5K1α		FcγRIIIa and Cyclin A1	
*R* ^2^	*P*‐value	*R* ^2^	*P*‐value
0.480	<0.000	0.334	0.001

### 3.2 FcγRIIIa expression in PCa cells is involved in tumor growth

To validate the presence of FcγRIIIa expression in PCa cells, we used castration‐resistant PCa cell line, PC‐3 cells to examine the FcγRIIIa mRNA expression. Since cancer cells utilize inflammatory myeloid cells to stimulate growth signaling in cancer cell, we cocultured PC‐3 cells with monocytes U‐937 cells to examine the expression of FcγRIIIa in PC‐3 cells after being cocultured with U‐937 cells. FcγRIIIa mRNA expression was observed in PC‐3 cells and U‐937 cells cultured alone as well as in PC‐3 cells from the coculture with U‐937 cells as determined by the semiquantitative RT‐PCR analysis using the primers specific for *FCGR3A* gene (Fig. S4A). The sequence of the PCR product of *FCGR3A* from PC‐3 cells exhibited 100% match with the consensus sequence of *FCGR3A* from NCBI database (Fig. S4B). Next, we examined FcγRIIIa protein expression in PC‐3 cells along with various types of PCa cell lines by using immunoblot analysis. Interestingly, FcγRIIIa protein exhibited high level in the castration‐resistant cell lines including C4‐2, VCaP, PC‐3 and PC‐3M cells, while its expression level appeared to be relatively low in the nonmalignant PNT1A cells (Fig. S5).

**Fig. 2:**
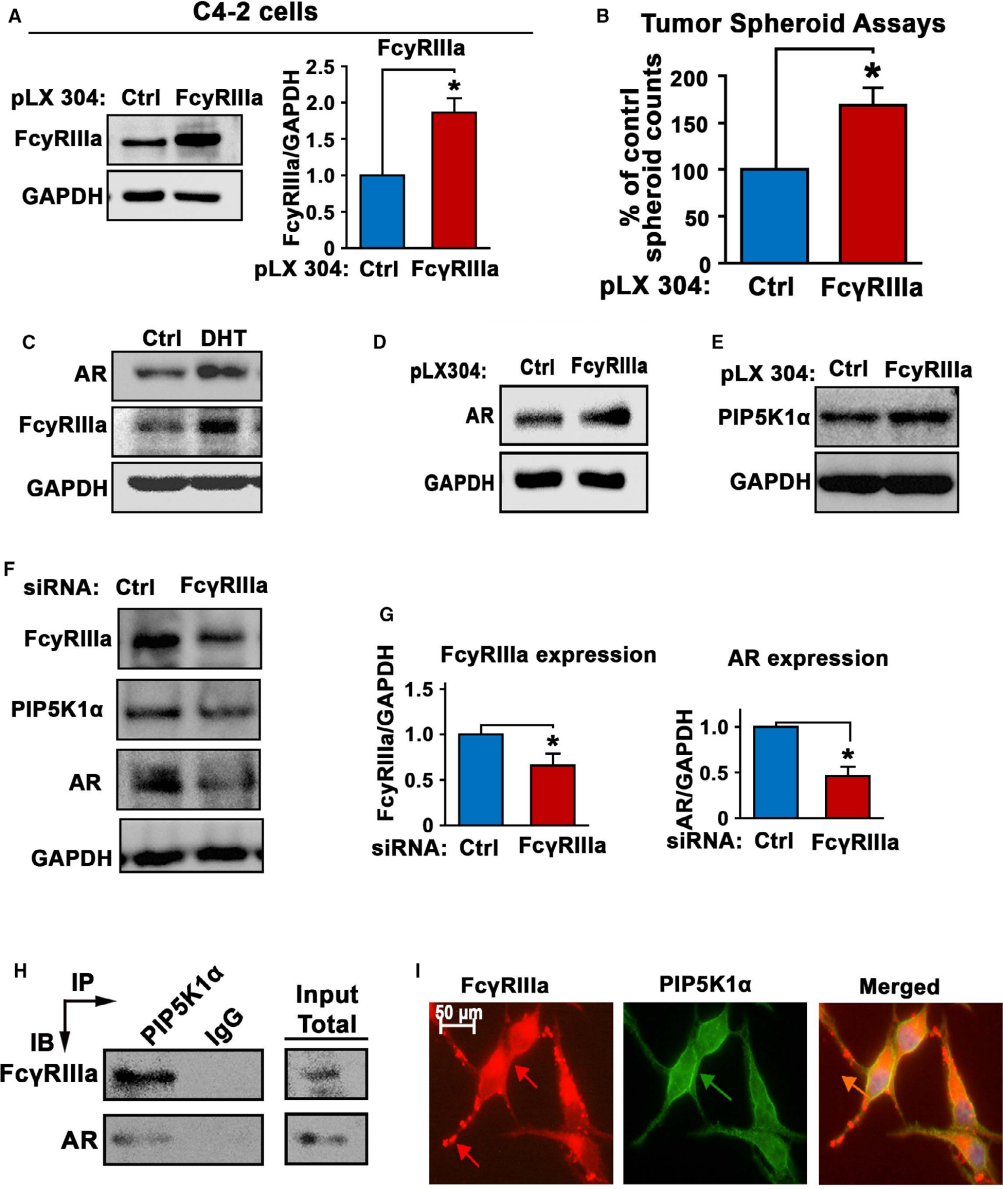
The role of FcγRIIIa in promoting tumorigenesis and its association with AR and PIP5K1α in C4‐2 cells. (A) Immunoblot analysis was performed to confirm the induced overexpression of FcγRIIIa in C4‐2 cells that were transfected with pLX304‐FCGR3A vector (FcγRIIIa) as compared with C4‐2 cells that were transfected with pLX304 control vector (Ctrl). The quantifications of the immunoblots are shown in the right panels. Data are presented as average of three independent experiments (*n* = 3), *P < *0.05, as indicated by ‘*’. The error bars indicate SEM. The student *t*‐test was used to determine the significance. (B) The effect of FcγRIIIa overexpression on the tumorigenic ability of C4‐2 cells was assessed using tumor‐spheroid formation assays. Data are presented as average of three independent experiments (*n* = 3), *P < *0.05, as indicated by ‘*’. The error bars indicate SEM. The student *t*‐test was used to determine the significance. (C) The effect of DHT treatment on FcγRIIIa and AR expression in C4‐2 cells was examined by using immunoblot analysis. C4‐2 cells treated with 0.1% DMSO as vehicle control (Ctrl) and C4‐2 cells treated with DHT at 5 nm (DHT) for 12 h are indicated. Data are representative of two independent experiments (*n* = 2) with each experiment performed in triplicates (*n* = 3). (D and E) The effect of FcγRIIIa overexpression on AR and PIP5K1α expression in C4‐2 cells was assessed using immunoblot analysis. The data are representative of three independent experiments (*n* = 3). (F) The effect of siRNA‐mediated knockdown of FcγRIIIa on AR and PIP5K1α expression in C4‐2 cells was assessed using immunoblot analysis. C4‐2 cells transfected with siRNA scramble control (ctrl) or siRNA to FcγRIIIa (FcγRIIIa) are indicated. Three independent experiments (*n* = 3) were performed. (G) The quantifications of the immunoblots of FcγRIIIa and AR are shown. Data are presented as average of three independent experiments (*n* = 3), *P < *0.05, as indicated by ‘*’. The error bars indicate SEM. The student *t*‐test was used to determine the significance. (H) The formation of protein complexes among FcγRIIIa, PIP5K1α, and AR was assessed by using immunoprecipitation (IP) assay. C4‐2 cells were subjected to immunoprecipitation (IP) assay in which antibody against PIP5K1α was used to pull down the immuno‐complexes, and antibody to IgG was used as a negative control. Antibodies against FcγRIIIa and AR were used for immunoblot analysis (IB). The equal amount of total lysates was used as input control for immunoblot analysis of the immuno‐precipitated protein lysates. Data are representative of at least two independent experiments (*n* = 2). (I) Immunofluorescence analysis was performed to assess the subcellular localization and its colocalization with PIP5K1α. Representative images of the subcellular localizations of FcγRIIIa expression (red), PIP5K1α (green), and overlapped image (Merged) are shown. The scale bar: 20 µm is indicated. Data are representative of four independent experiments (*n* = 4).

Since FcγRIIIa expression is significantly elevated in metastatic PCa tissues compared with the primary PCa tumors, we wanted to elucidate the role of FcγRIIIa in PCa progression. To this end, we transfected C4‐2 cells with a full‐length FcγRIIIa expressing plasmid or a control vector to induce FcγRIIIa overexpression. Overexpression of FcγRIIIa was confirmed by immunoblot analysis (Fig. 2A). To examine the effect of FcγRIIIa overexpression on tumor growth, we subjected C4‐2 cells that expressed FcγRIIIa or control vector to the 3‐D tumor spheroid assays. We found that C4‐2 cells overexpressing FcγRIIIa gave rise to higher numbers of tumor spheroids than that of controls (*P* = 0.0057; Fig. 2B). This suggests that elevated level of FcγRIIIa in PCa cells led to increased ability of tumorigenesis of PCa cells *in vitro*. Since steroid hormone DHT promotes growth of PCa by inducing activation of AR pathways that are associated with PCa growth and invasion, we therefore examined the relationship between DHT/AR pathways and FcγRIIIa expression in PCa cells. We employed C4‐2 cell line to DHT treatment and examined the effect of DHT on FcγRIIIa expression. We found that DHT treatment at 5 nm resulted in increased FcγRIIIa expression, which was equivalent to its effect on induction of AR expression in C4‐2 cells (Fig. 2C). Thus, FcγRIIIa expression is responsive to androgen stimulation in PCa cells. As mentioned above, we found a significant correlation between FcγRIIIa and PIP5K1α in primary tumor and metastatic lesions from PCa patients. Given that PIP5K1α acts on upstream of AR pathways, we next investigated the relationship between FcγRIIIa and AR, as well as the interaction between FcRIIIa and PIP5K1. Induced overexpression of FcγRIIIa led to a slight increase in AR and PIP5K1α in C4‐2 cells compared with the controls. However, the statistically significance was not achieved (Fig. 2D,E). Next, we examined the effect of FcγRIIIa depletion on expression of AR and PIP5K1α in C4‐2 cells. FcγRIIIa was silenced via siRNA‐mediated knockdown in C4‐2 cells. The significant downregulation of FcγRIIIa in C4‐2 cells compared with that of siRNA control was confirmed using immunoblot analysis (*P* = 0.024; Fig. 2F,G). We found that silence of FcγRIIIa led to the downregulation of PIP5K1α and AR expression as compared with that of siRNA controls in C4‐2 cells (for PIP5K1α, *P* = 0.005; for AR, *P* = 0.001; Fig. 2F,G). Thus, FcγRIIIa depletion has significant effect on the expression of PIP5K1α and AR, suggesting that FcγRIIIa is required by PCa cells to regulate PIP5K1α and AR expression.

To further determine the relationship among FcγRIIIa, AR, and PIP5K1α in PCa cells, we performed immunoprecipitation assays to examine whether FcγRIIIa may form protein–protein complexes with AR and PIP5K1α in C4‐2 cells. We found that both FcγRIIIa and AR were present in the immuno‐complexes that are associated with PIP5K1α (Fig. 2H). This suggests that FcγRIIIa is able to form protein complexes with PIP5K1α and AR as well. Thus, FcγRIIIa is functionally linked to AR and PIP5K1α via protein–protein interaction. Immunofluorescent analysis was performed to examine the subcellular localization of FcγRIIIa in C4‐2 cells. We found that FcγRIIIa expression was highly enriched in the membrane/cytoplasmic compartments, and appeared to be colocalized with PIP5K1α in the membrane compartment of C4‐2 cells (Fig. 2I).

### 3.3 The functional association between AR and FcγRIIIa in PCa cells

**Fig. 3:**
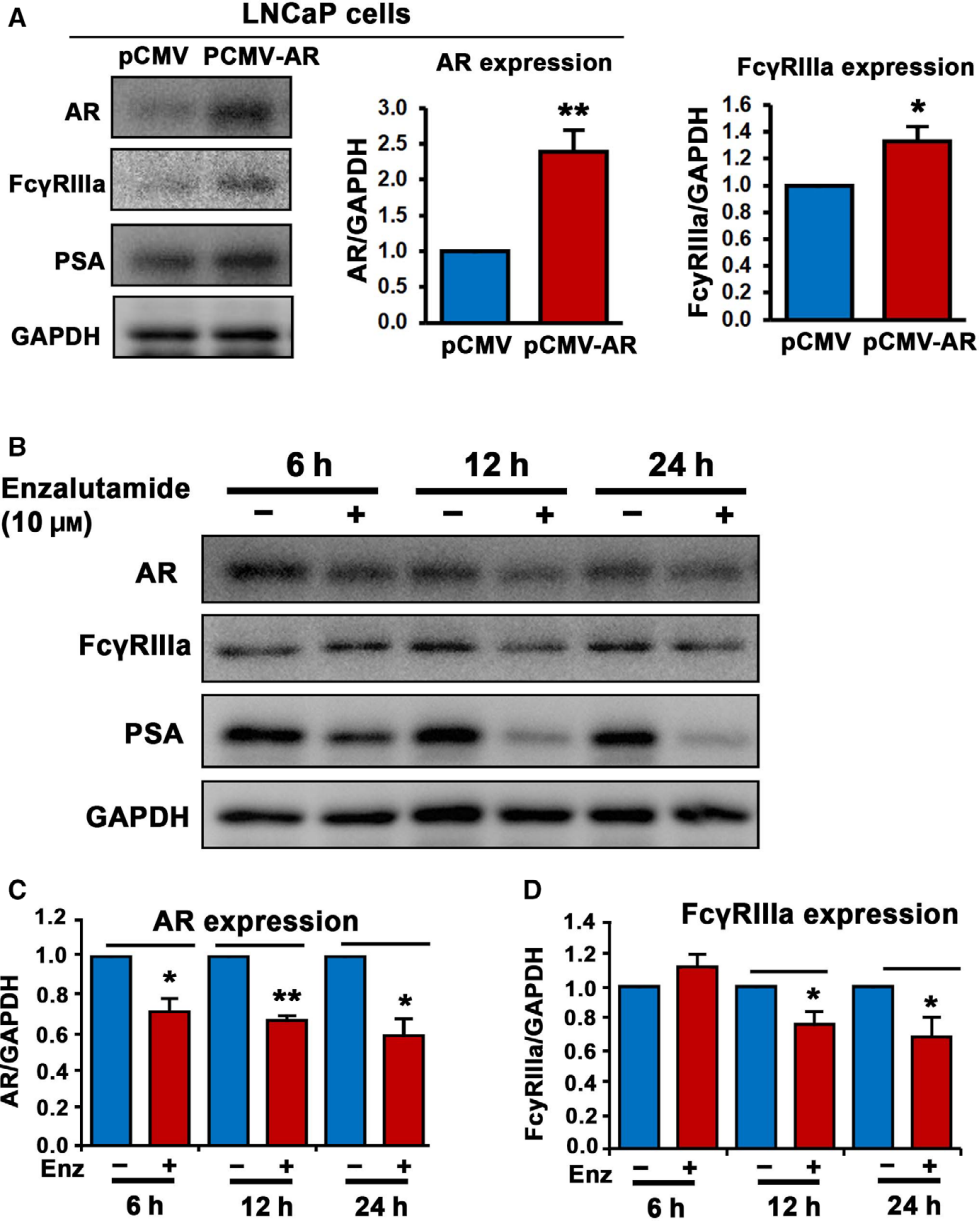
The association between AR and FcγRIIIa in LNCaP cells. (A) The effect of elevated level of AR expression on FcγRIIIa and PSA protein expression in LNaCp cells was assessed using immunoblot analysis. The quantifications of the immunoblots for AR and FcγRIIIa are shown in the right panels. Expression of AR and FcγRIIIa was significantly higher in LNCaP cells transfected with PCMV‐AR vector than that of control pCMV vector, for AR, *P* = 0.003, for FcγRIIIa, *P* = 0.03. Data are presented as average of three independent experiments (*n* = 3), **P < 0.01 and *P < 0.05 are indicated. The error bars indicate SEM. The student *t*‐test was used to determine the significance. (B) The effect of inhibition of AR using enzalutamide on FcγRIIIa expression in LNCaP cells was assessed using immunoblot analysis. Data are representative of two independent experiments (*n* = 2) with each experiment performed in duplicates (*n* = 2). (C and D) The quantifications of the immunoblots for AR and FcγRIIIa are shown. Data are presented as average of two independent experiments (*n* = 2) with each experiment performed in duplicates (*n* = 2), *P < *0.05, as indicated by ‘*’. *P < *0.01 is indicated by ‘**’. Expression of AR and FcγRIIIa was significantly decreased in LNCaP cells treated with enzalutamide for 12 h and 24 h, respectively, as compared with that of vehicle control‐treated cells (for AR, enzalutamide treatment vs. control treatment for 6 h, *P* = 0.026; 12 h, *P* = 0.003; and 24 h, *P* = 0.021; for FcγRIIIa, enzalutamide treatment vs. control treatment for 6 h, *P* = 0.325; 12 h, *P* = 0.03; and 24 h, *P* = 0.034.). The error bars indicate SEM. The student *t*‐test was used to determine the significance.

To gain deeper understanding of the mechanisms underlying the association between FcγRIIIa and AR in PCa cells, we examined the effect of AR overexpression on FcγRIIIa expression at protein and mRNA levels. AR overexpression was induced in LNCaP cells by transfecting the cells with a vector carrying full‐length AR or a control vector. We found that elevated expression of AR resulted in a significant increase in FcγRIIIa protein expression in LNCaP cells as compared with that of control (*P* = 0.03; Fig. 3A). However, AR overexpression had no significant effect on FcγRIIIa mRNA expression in LNCaP cells, as determined by using semiquantitative RT‐PCR analysis (Fig. S6). Since enzalutamide is an inhibitor for AR and it targets ligand‐binding domain of AR, resulting in inhibition of AR activity. We therefore examined whether inhibition of AR using enzalutamide might have a direct effect on FcγRIIIa protein and mRNA expression. To this end, we treated LNCaP cells with enzalutamide for 6 h, 12 h, and 24 h, respectively. We then examined the effect of AR inhibition by enzalutamide on the expression of AR, FcγRIIIa, and PSA, a known downstream target of AR. As expected, enzalutamide treatment resulted in significant decrease in AR expression after 6 h of treatment and throughout 12 to 24 h (for AR, enzalutamide treatment vs. control treatment for 6 h, *P* = 0.026; 12 h, *P* = 0.003; and 24 h, *P* = 0.021; Fig. 3B,C,D). Interestingly, enzalutamide treatment led to a significant downregulation of FcγRIIIa protein expression readily after 12 h and throughout 24 h of post‐treatment (enzalutamide treatment vs. control treatment for 6 h, *P* = 0.325, for 12 h, *P* = 0.03 and for 24 h, *P* = 0.034; Fig. 3B–D). Enzalutamide did not appear to have significant effect on FcγRIIIa mRNA expression in LNCaP cells as measured by quantitative RT‐PCR (Fig. S7). The effect of AR inhibition on FcγRIIIa readily appeared at early time point of 12 h, and LNCaP is a slow proliferating PCa cell line with a doubling time at approximately 60 h; these findings suggest that AR and FcγRIIIa may be directly under each other’s control.

### 3.4 The role of FcγRIIIa in AR‐independent castration‐resistant PC‐3 cells

**Fig. 4:**
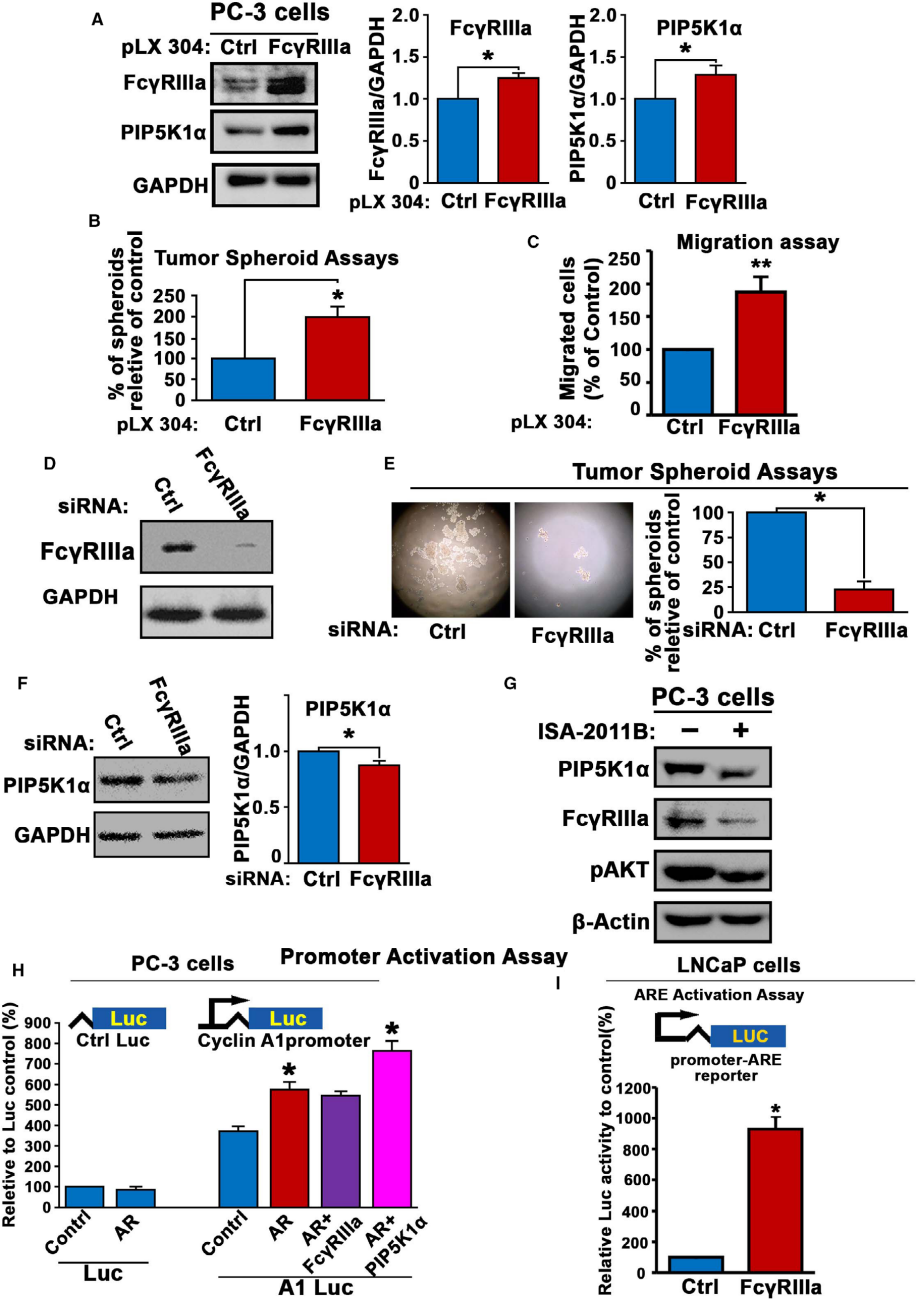
The role of FcγRIIIa in promoting tumorigenesis and its association with AR and PIP5K1α in PC‐3 cells. (A) The effect of induced overexpression of FcγRIIIa on expression of PIP5K1α in PC‐3 was assessed by immunoblot analysis. The quantifications of the immunoblots are shown in the right panels. Expression of FcγRIIIa and PIP5K1α was significantly higher in PC‐3 cells transfected with FcγRIIIa than that of control vector, for PIP5K1α, *P* = 0.011. Data are representative of three independent experiments (*n* = 3), *P < *0.05, as indicated by ‘*’. The error bars indicate SEM. The student *t*‐test was used to determine the significance. (B) The effect of FcγRIIIa overexpression on the tumorigenic ability of PC‐3 cells was assessed using tumor‐spheroid formation assays. Data shown are representative of two independent experiments (*n* = 2) with each experiment performed in triplicates (*n* = 3), *P < *0.05, as indicated by ‘*’. The error bars indicate SEM. The student *t*‐test was used to determine the significance. (C) The effect of induced overexpression of FcγRIIIa on migratory ability of PC‐3 cells was assessed by using migration assay. Data shown are presented as average of three independent experiments (*n* = 3), *P < *0.01, as indicated by ‘**’. The error bars indicate SEM. The student *t*‐test was used to determine the significance. (D) The depletion of FcγRIIIα in PC‐3 cells transfected with siFcγRIIIa RNA (FcγRIIIa) compared with PC‐3 cells transfected with siRNA scramble control (Ctrl) was assessed by using immunoblot analysis. Data are representative of two independent experiments (*n* = 2) with each experiment performed in duplicates (*n* = 2). (E) The effect of FcγRIIIa knockdown on tumorigenic ability of PC‐3 cells was determined using tumor‐spheroid formation assays. Representative images of tumor spheroids are shown. The spheroid counts are shown in the right panel. Mean tumor‐spheroid counts in si‐control and si‐FcγRIIIa PC‐3 cells were 56 and 14, difference = 42, 95% CI in si‐control = 43‐68 and si‐FcγRIIIa = 6‐21, *P* = 0.012. Data are representative of two independent experiments (*n* = 2), and each experiment was performed in triplicates (*n* = 3), *P < *0.05, as indicated by ‘*’. The error bars indicate SEM. The student *t*‐test was used to determine the significance. (F) The effect of FcγRIIIa knockdown on PIP5K1α in PC‐3 cells was determined using immunoblot analysis. Data shown in the right panel are representative of three independent experiments (*n* = 3), *P < *0.05, as indicated by ‘*’. The error bars indicate SEM. The student *t*‐test was used to determine the significance. (G) The effect of inhibition of PIP5K1α by its inhibitor ISA‐2011B on IP5K1α, FcγRIIIa, and pAKT in PC‐3 cells was determined using immunoblot analysis. Data are representative of at least three independent experiments (*n* = 3). (H) Effect of induced AR alone or together with induced FcγRIIIa or PIP5K1α expression on the activity of full‐length cyclin A1 promoter was assessed using luciferase assay. The vectors were induced together with luc‐reporter vector ‘Luc’ or cyclin A1 promoter‐luc‐reporter vector ‘A1‐Luc’ into PC‐3 cells (For AR alone, *P* = 0.003, for AR+PIP5K1α, *P = *0.008). Data are representative of at least two independent experiments (*n* = 2), and each experiment was performed in triplicates (*n* = 3). The error bars indicate SEM. The student *t*‐test was used to determine the significance. (I) Effect of FcγRIIIa overexpression on androgen‐responsive (ARE) promoter activity in LNCaP cells was carried out using the dual‐luciferase assays. FcγRIIIa overexpression‐induced ARE reporter luciferase activity led to an increase by 100% relative to controls in LNCaP cells, *P* = 0.013. Data shown are presented as average of two independent experiments (*n* = 2), and each experiment was performed in triplicates, *P < *0.05, as indicated by ‘*’. The error bars indicate SEM. The student *t*‐test was used to determine the significance.

To further elucidate the role of FcγRIIIa and the underlying cellular mechanisms in PCa growth and progression, we employed PC‐3 cells that lack functional AR, which represent an ideal model to assess the relationship between FcγRIIIa and PIP5K1α. To do this, FcγRIIIa overexpression was induced in PC‐3 cells by transfecting the cells with a vector carrying full‐length FcγRIIIa or a control vector, and FcγRIIIa overexpression was verified by immunoblot analysis (Fig. 4A). We found that elevated expression of FcγRIIIa resulted in a significant increase in PIP5K1α expression as compared with the control (*P* = 0.02; Fig. 4A). The effect of FcγRIIIa overexpression on growth of PC‐3 tumor was determined using tumor‐spheroid assays. Similar to what was observed in C4‐2 cells, FcγRIIIa overexpression resulted in increased ability of PC‐3 cells to form tumor spheroids as compared with the controls (*P* = 0.001; Fig. 4B). This data suggest that FcγRIIIa overexpression is able to promote tumorigenesis in PC‐3 cell model.

To elucidate the role of FcγRIIIa in PCa progression, we assessed the effect of FcγRIIIa overexpression on migratory ability of PC‐3 cells. PC‐3 cells that overexpressed FcγRIIIa displayed significantly higher migratory ability compared with the control (*P* < 0.001; Fig. 4C).

To further assess the functional importance of FcγRIIIa in tumor progression, we silenced FcγRIIIa using siRNA‐mediated knockdown. PC‐3 cells that were transfected with siRNA to FcγRIIIa or control siRNA were subjected to the tumor‐spheroid formation assays. In contrast to the effect of FcγRIIIa overexpression on tumor‐spheroid formation, silence of FcγRIIIa led to remarkable decrease in ability of PC‐3 cells to form tumor spheroids relative to that of controls (*P* = 0.012; Fig. 4D,E). This suggests that elevated level of FcγRIIIa is functional important for PCa cells to gain tumorigenic ability. Similar to what was observed in C4‐2 cells, siRNA‐mediated knockdown of FcγRIIIa led to a significant downregulation of PIP5K1α expression (*P* = 0.011; Fig. 4F). Next, we examined the effect of inhibition of PIP5K1α on FcγRIIIa in PC‐3 cells. We applied a selective inhibitor of PIP5K1α, ISA‐2011B and examined ISA‐2011B on FcγRIIIa in PC‐3 cells. Interestingly, inhibition of PIP5K1α by ISA‐2011B resulted in decreased FcγRIIIa expression, which was coincident with the downregulation of pAKT induced by ISA‐2011B in PC‐3 cells as compared with controls (Fig. 4G). Similar to what was shown in Fig. 3, these data also show that PIP5K1α and FcγRIIIa mutually affect each other, which further support our observation on that PIP5K1α and FcγRIIIa form protein–protein complexes, as mentioned in Fig. 2. PIP5K1α promotes prostate cancer cell survival and invasion through regulation of expression of AR in PCa cells [28, 29]. To this end, we wanted to investigate whether FcγRIIIa and PIP5K1α might act as coregulators of AR to enhance transcriptional activity of AR on its target genes, and we utilized a cyclin A1 full‐length promoter‐luciferase reporter construct as described [41] and examined the effect of FcγRIIIa and PIP5K1α on AR transcriptional activity on its target gene cyclin A1 in PC‐3 cells. AR alone increased remarkably cyclin A1‐luciferase activity as compared with that of controls (*P* = 0.003; Fig. 4H). FcγRIIIa had no additive effect on AR to further enhance cyclin A1 promoter activation (Fig. 4H). Interestingly, PIP5K1α and AR in combination increased remarkably cyclin A1‐luciferase activity as compared to that of AR alone (*P* = 0.008; Fig. 4H). These data suggest that FcγRIIIa may serve as a coregulator of AR via PIP5K1α. To further elucidate the functional impact of FcγRIIIa on the downstream target genes in PCa cells, we employed androgen‐dependent LNCaP cells and carried out dual*‐*luciferase assays by using androgen‐responsive (ARE) luciferase reporter construct. FcγRIIIa overexpression induced ARE reporter luciferase activity, which led to an increase in ARE promoter activity by 100% relative to controls in LNCaP cells (*P* = 0.013; Fig. 4I). Thus, FcγRIIIa is able to mediate the transcriptional activity of the key factors that contribute to PCa progression.

### 3.5 Targeted inhibition of FcγRIIIa in PC‐3M cells reduced tumor growth in xenograft mice

**Fig. 5:**
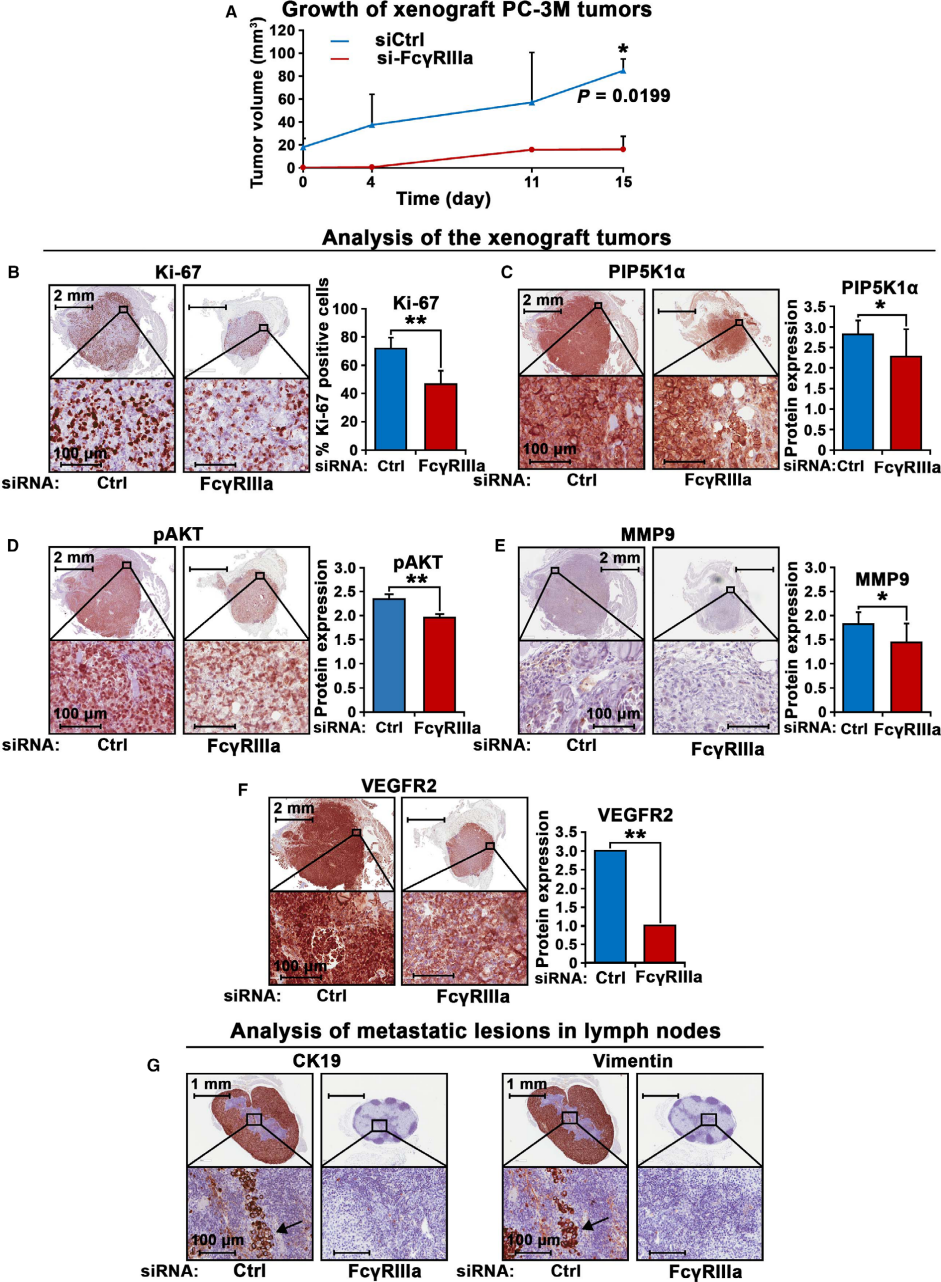
The inhibitory effect of FcγRIIIa knockdown on growth of tumors in xenograft mouse model. (A) Growth curves of PC‐3 M tumors expressing si‐control RNA (siCtrl) or si‐ FcγRIIIa (FcγRIIIa) in xenograft mice are shown (*n* = 4 per group). Tumor volumes are indicated in Y‐axis, and the measurement days are indicated in x‐axis. Tumors from each group were collected at the end of the experiment. (B, C, D, E, F) Expression of the key proteins for proliferation and invasion in the xenograft tumors collected from the mice was assessed by using immunohistochemical analysis. Representative images of the siCtrl tumors and si‐FcγRIIIa tumors that were stained with antibodies against Ki‐67, PIP5K1α, pAKT, MMP9, and VEGFR2 are shown. Quantification of the staining intensity of the proteins is shown in the right panels. Mean Ki‐67‐positive cells in siCtrl and siFCGR3A tumors were 71.82% and 46.67%, difference = 25.15%; 95% CI = 38.40 to 54.93%, *P* < 0.001. Mean PIP5K1α expression in si‐control and si‐FcγRIIIa tumors was 2.82 and 2.20, difference = 0.54, 95% CI for si‐control = 2,78–2,85, si‐FcγRIIIa = 2.13‐2.43. *P* = 0.048; mean pAKT expression for si‐control and si‐FcγRIIIa were 2.34 and 1.96, difference = 0.39, 95% CI for si‐control = 2.3–2.38, and for si‐FcγRIIIa = 1.94–1.98, *P* = 0.0045. Mean MMP9 expression in si‐control and si‐FcγRIIIa was 1.82 and 1.44, difference=0.37, 95% CI in si‐control = 1.8‐1.84, and in si‐FcγRIIIa = 1.39‐1.5, *P* = 0.028. **P < 0.01 and *P < 0.05 are indicated. Tumors from the two groups (for si‐control group, *n* = 3; for si‐FcγRIIIa group, *n* = 2) were stained with the indicated antibodies and were evaluated. The error bars indicate SEM. The student *t*‐test was used to determine the significance. The scale bars: 2 mm and 100 µm in the images in B, C, D, E, and F are indicated. (G) Representative images of the lymph nodes containing metastatic lesions from the xenograft mice bearing si‐FcγRIIIa tumors as compared with that of si‐control RNA (siCtrl) are shown. Tumors were immune‐stained with the antibodies against CD19 and Vimentin that are markers for cancer cells. Tumor cells positive to the markers were indicated by the arrows. Tumors from the two groups (for si‐control group, *n* = 3; for si‐FcγRIIIa group, *n* = 2) were stained with the indicated antibodies and were evaluated. The scale bars: 1 mm and 100 µm are indicated.

We have previously reported that PC‐3M cells are able to initiate metastasis to distant organs in xenograft mice [40]. We therefore employed PC‐3M xenograft tumor models in mice to elucidate the role of FcγRIIIa in PCa progression. To this end, we silenced FcγRIIIa in PC‐3M cells by using siRNA‐mediated knockdown. We then implanted subcutaneously equal amount of si‐FcγRIIIa PC‐3M cells and si‐control PC‐3M cells into the nude mice. The growth of PC‐3M tumors in xenograft mice was measured and monitored. At the end of experiments, the mean tumor volumes in mice that received si‐FcγRIIIa PC‐3M cells were significantly smaller than that of controls (*P* = 0.020; Fig. 5A). We then assessed expression of the key marker proteins including Ki‐67, phosphorylated AKT, MMP9, and VEGFR2 that control proliferation and invasiveness of PCa cells. Consistent with what was observed on the tumor volumes, si‐FcγRIIIa PC‐3M tumors displayed a significantly reduced proliferation rate relative to controls, as determined by using Ki‐67 staining (*P* < 0.001; Fig. 5B). Similarly, we found that expression of PIP5K1α, pSer‐473AKT, MMP9, and VEGFR2 was significantly downregulated in si‐FcγRIIIa PC‐3M tumors compared with that of si‐control tumors, which was coincident with the reduced volumes of si‐FcγRIIIa PC‐3 M tumors (for PIP5K1α expression, *P* = 0.048; for pAKT, *P* = 0.0045; for MMP9 expression, *P* = 0.028; and for VEGFR2, *P* < 0.001; Fig. 5C–F). We have previously reported that PC‐3M cells are able to initiate metastasis to distant organs in xenograft mice [40]. We therefore examined the apparent metastasis in the lymph nodes in mice that have received si‐FcγRIIIa PC‐3M or si‐control PC‐3M cells. We found that mice bearing si‐control PC‐3M tumors had lymph node metastasis, whereas mice bearing si‐FcγRIIIa tumors were free of lymph node metastasis (Fig. 5G). Further, si‐control PC‐3M tumors expressed cytokeratin 19 (CK19) and vimentin, the human epithelial cell markers. In contrast, si‐FcγRIIIa tumors were negative to CK19 and vimentin expression. These data suggest that inhibition of FcγRIIIa greatly reduced growth and metastatic potentials of primary tumors in xenograft mouse models. It is likely that FcγRIIIa promotes PCa growth and invasion via its downstream PIP5K1α/AKT and VEGFR2 signaling pathways.

### 3.6 Inhibitory effect of anti‐FcγRIIIa antibody on PCa growth in PCa cell line models and in PCa xenograft mice

**Fig. 6:**
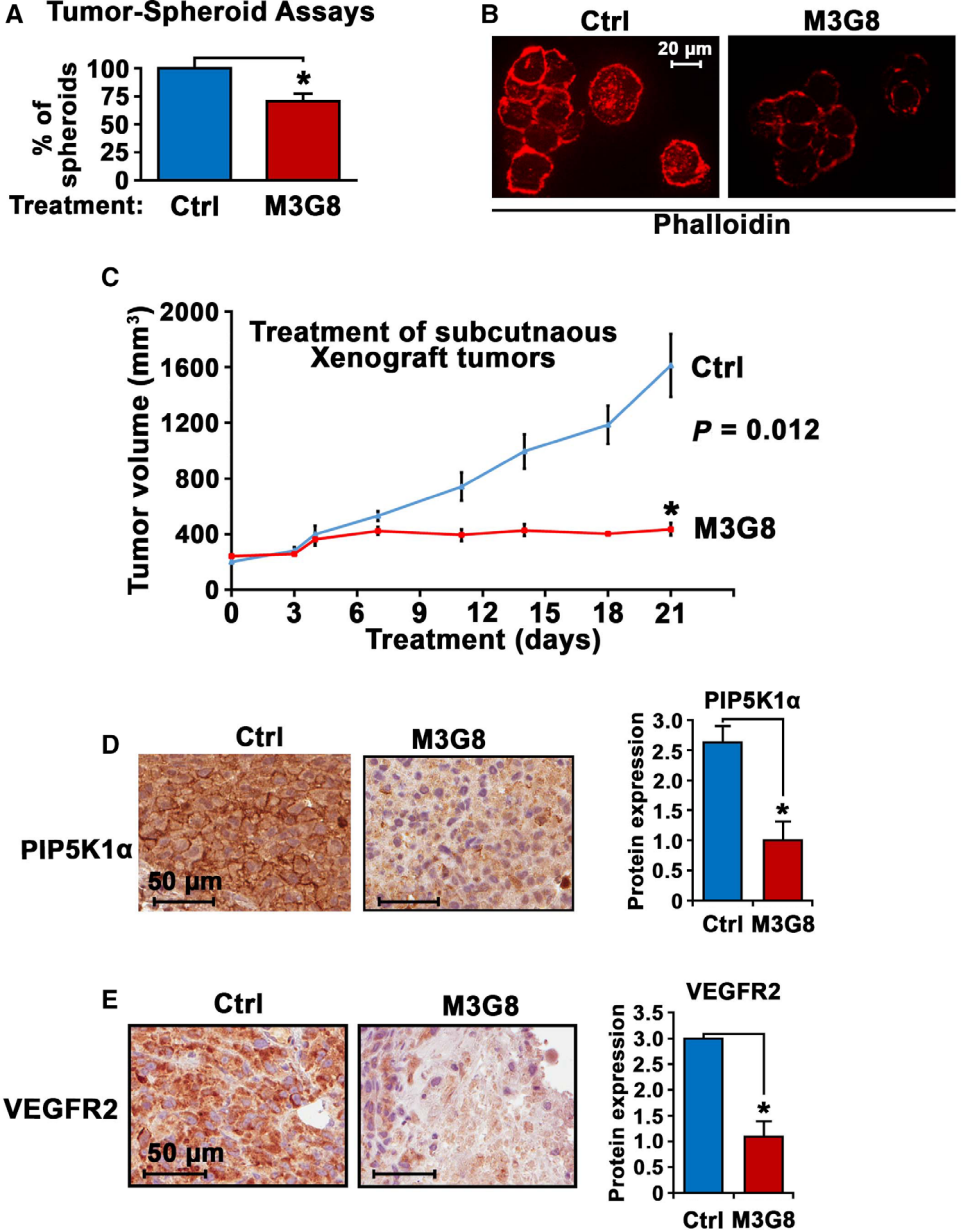
The interlink between PIP5K1α and FcγRIIIa and inhibition of via monocloncal antibody M3G8 in PCa cells. (A) The effect of M3G8 on growth of tumor spheroids derived from PC‐3 cells cocultured with U‐937 cells. Tumor spheroids were treated with control or M3G8 antibodies. Mean tumor‐spheroid counts in control antibody‐treated and in M3G8‐treated groups = 38‐26, difference = 12, 95%CI = 22–31, *P* = 0.028. Data are representative of two independent experiments (*n* = 2), and each experiment was performed in triplicates, *P < *0.05, as indicated by ‘*’. The error bars indicate SEM. The student *t*‐test was used to determine the significance. (B) Representative immunofluorescent images from (A) showing the tumor spheroids treated with M3G8 or control antibodies that are highlighted with phalloidin staining (red). The experiments were replicated (*n* = 2). The scale bar: 20 µm is indicated. (C) Growth of PC‐3 subcutaneous xenograft tumors that were treated with control IgG antibody (Ctrl) or M3G8 antibody. Treatment started on day 0 when the mean tumor volume reached to over 300 mm^3^ and ended on day 21 (*n* = 3‐4 mice per group). Y‐axis indicates tumor volumes, and x‐axis indicates days of treatment. *P* = 0.012. *P < 0.05 is indicated. (D and E) Immunohistochemical analysis of the xenograft tumors from mice treated with control antibody or M3G8 antibody. Representative microphotographs of images showing expression of PIP5K1α and VEGFR2 are shown in left panels and quantification of the staining intensity of the antibodies against these proteins in tumor cells are in shown in the right panels. Mean PIP5K1α expression in control‐treated and M3G8‐treated was 2.63 and 1.0, difference = 1.63, 95%Cl for control‐treated = 2.49‐2.76 and for M3G8‐treated = 0.72‐1.28, *P* = 0.01. Mean VEGFR2 expression in control‐treated and M3G8‐treated= 3.0 and 1.08, difference = 1.92, 95% Cl for M3G8‐treated = 0.77–1.4, *P* = 0.01). Tumors from the two groups (*n* = 3) were stained with the indicated antibodies and were evaluated. *P < 0.05 is indicated. The error bars indicate SEM. The student *t*‐test was used to determine the significance. The scale bars: 50 µm is indicated.

To further study the role and cellular mechanisms of FcγRIIIa in tumor growth and invasion, we examined the antitumor effect of anti‐FcγRIII antibody (M3G8) in *in vitro* and *in vivo* systems. We subjected PC‐3 cells to the formation of tumor spheroids. The tumor spheroids were then subjected to the treatment with M3G8 or control antibody. We observed that M3G8 treatment led to a remarkably reduced number of tumor spheroids as compared with controls (*P* = 0.028; Fig. 6A). Also, there was a pronounced alteration in cell–cell contacts and a reduced phalloidin staining in tumor spheroids treated with M3G8 compared with that of controls (Fig. 6B).

Next, we wanted to investigate whether blockade of FcγRIIIa using purified anti‐human FcγRIII monoclonal antibody termed M3G8 may suppress growth of PCa tumors in xenograft mice. To this end, we established xenograft mice bearing subcutaneously implanted PC‐3 tumors, which were less invasive, but grow rapidly as compared to PC‐3M tumors. PC‐3 tumors were allowed to grow into approximately 300 mm^3^ in size and were randomized into two groups. The two groups of mice were treated with M3G8 or control antibody. The M3G8‐treated group had tumors which were fourfold smaller in size relative to the control group after treatment for 21 days (mean volume of tumors in control group and M3G8 group was 1612 mm^3^ and 436 mm^3^, respectively, difference = 1176 mm^3^; 95% CI = 384‐486, *P* < 0.01; Fig. 6C). This was consistent with the inhibitory effect of targeted inhibition of FcγRIIIa on PC‐3M tumors shown above. Immunohistochemical analysis of tumor tissues further revealed that M3G8‐treated tumors exhibited reduced expression of PIP5K1α and VEGFR2 as compared to that of controls (for PIP5K1α, *P* = 0.01; for pAKT, *P* = 0.02; and for VEGFR2, *P* = 0.01; Fig. 6D,E).

### 3.7 Inhibitory effect of anti‐FcγRIIIa antibody on PCa growth and metastasis in mice

**Fig. 7:**
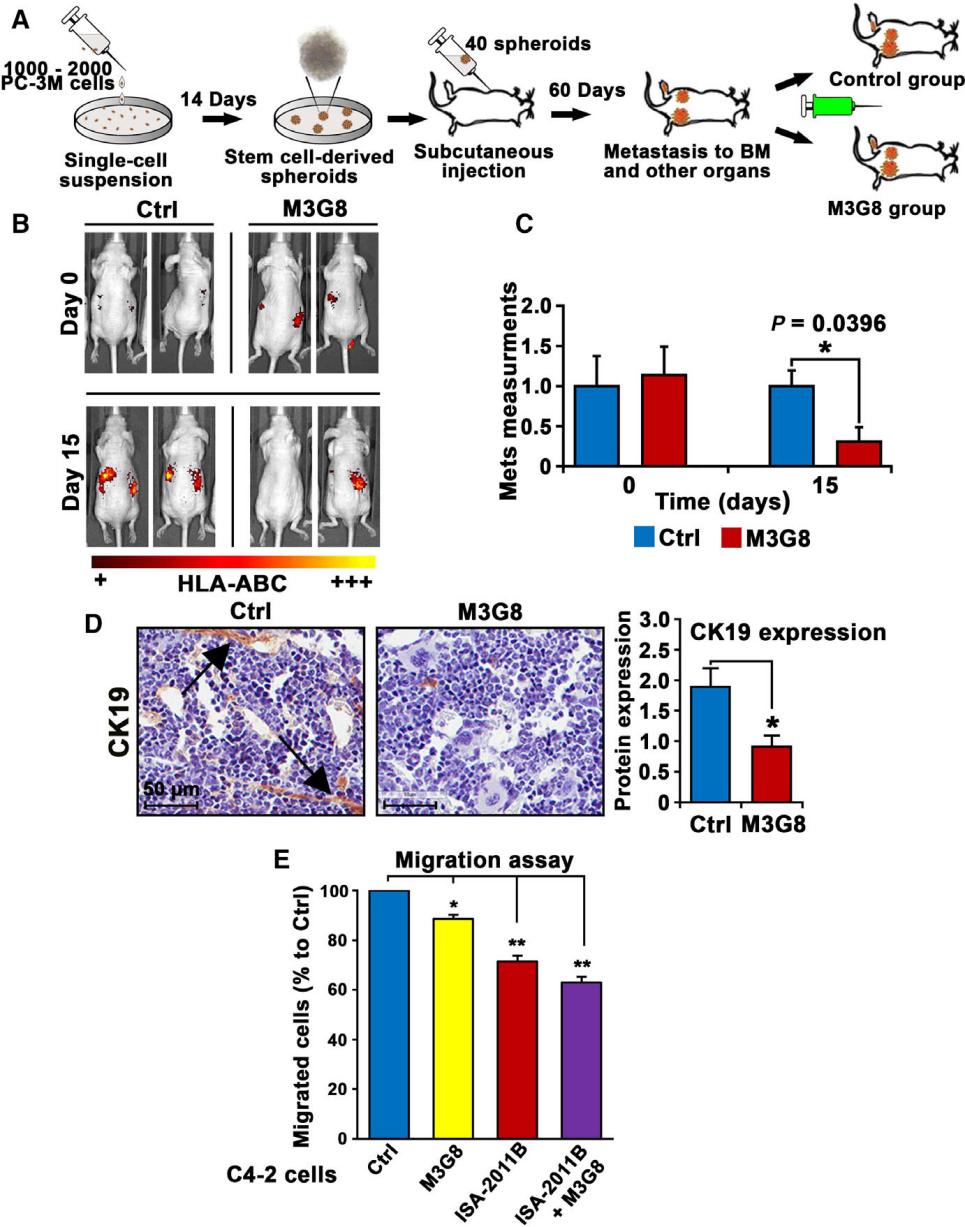
The effect of M3G8 on tumor growth and metastasis in xenograft mouse models. (A) Schematic illustration depicts the experimental procedures of establishment of distant metastasis including bone metastasis by using tumor spheroids derived from ALDH^high^ PC‐3 M cells, and *in vivo* treatment regimens using M3G8 antibody. (B) Representative images to show the bioluminescent *in vivo* imaging on visualization of tumor metastasis in mice bearing metastasis and were treated with M3G8 or control antibodies. The signals were obtained by using fluorochrome‐conjugated HLA‐ABC antibody which was injected into the mice 6 h before applying mice on the IVIS imaging device. (C) Mean metastatic areas and intensity in pixels for Ctrl group (*n* = 4) and M3G8 group (*n* = 3) are shown. On day 0, *P* = 0.8257. On day 15 post‐treatment, mean value of metastatic areas plus signal intensity for control‐treated and M3G8‐treated groups was 250 and 76, difference = 174, 95% CI for control group = 185‐310, and for M3G8 group = 0–157, *P* = 0.039. The error bars indicate SEM. The student *t*‐test was used to determine the significance. (D) Representative microphotographs showing CK19‐positive cells in the bone marrow, indicating bone metastasis. Quantification of the staining intensity of CK19 in the bone marrow of the mice (*n* = 2–3) that were treated with Ctrl or M3G8 is shown in the right panel. The arrow heads point to the CK19‐positive tumor cells or tumor cell clusters. *P < 0.05 is indicated. The student *t*‐test was used to determine the significance. The scale bar: 50 µm is indicated. (E) The effect of M3G8 and ISA‐2011B alone or in combination on the migratory ability of C4‐2 cells was assessed by using the migration assays. After treatment with the agents, the equal amount of the cells from different groups was subjected to the Boyden chamber migration assay for 18 h. M3G8 treatment or ISA‐2011B treatment alone reduced migratory ability of C4‐2 cells as compared with that of controls (for M3G8, *P* = 0.016; for ISA‐2011B, *P* = 0.006). Combination of M3G8 and ISA‐2011B reduced the migratory ability of the cells as compared with that of controls (*P* = 0.003). Data are presented as average of two independent experiments (*n* = 2). **P < 0.01 and *P < 0.05 are indicated. The error bars indicate SEM. The student *t*‐test was used to determine the significance.

Next, we wanted to investigate whether blockade of FcγRIIIa using M3G8 may reduce/inhibit distant metastasis of PCa. We have previously reported that ALDH^high^ stem‐like subpopulations isolated from PC‐3M cells initiated metastatic growth in distant organs such as bone/bone marrow in xenograft mice [40]. To this end, we sorted stem‐like ALDH^high^ subpopulations of PC‐3M cells using FACS‐based ALDEFLUOR assay and subjected the stem‐like ALDH^high^ subpopulations to the formation of 3‐dimensional tumor spheroid (Fig. 7A). The tumor spheroids were then implanted subcutaneously into the nude mice (40 tumor spheroids/mouse) to allow formation of distant metastasis (Fig. 7A). Virtually all mice that received tumor spheroids had developed distant metastasis 60 days postimplantation, as measured and quantified by using *in vivo* imaging assays as described previously [40] (Fig. 7B,C). Mice bearing metastatic lesions were randomized into two groups and were treated with intraperitoneal injection of M3G8 or control antibody at 5 mg·kg^−1^ dose (Fig. 7B,C). At the end of the experiments, there was a significant reduction in metastatic burdens in mice treated with M3G8 compared with that of control, as quantified using *in vivo* imaging analysis (*P* = 0.039; Fig. 7B,C). M3G8 treatment did not induce weight loss or other detectable adverse events in the mice. There was a significant higher proportion of cells positive to cytokeratin 19 (CK19), a marker of human epithelial cell origin, in the bone marrows from xenograft mice treated with control antibody compared with those treated with M3G8 (*P = *0.02; Fig. 7D). These data suggest that inhibition of FcγRIIIa inhibits metastatic growth of PCa cells in distant organs in xenograft mice.

To test the therapeutic potentials of combination therapies of M3G8 and ISA‐2011B, we assessed the effects of M3G8, ISA‐2011B alone or in combination on the invasiveness of C4‐2 cells. C4‐2 cells that were treated with M3G8 or ISA‐2011B alone or in combination were subjected to the migration assays. Similar to ISA‐2011B, M3G8 treatment alone significantly reduced migratory ability of C4‐2 cells (for M3G8, *P* = 0.016; for ISA‐2011B, *P* = 0.006; Fig. 7E). Combination of M3G8 and ISA‐2011B had greater inhibitory effect as compared to that of M3G8 alone on the migratory ability of C4‐2 cells (for combination of M3G8 and ISA‐2011B vs. control, *P* = 0.003; for combination of M3G8 and ISA‐2011B vs. M3G8, *P* = 0.011; Fig. 7E). These data suggest that combination treatment using M3G8 and ISA‐2011B may have an additive inhibitory effect on PCa cells.

## 4 Discussion

In this study, we discovered that the expression of FcγRIIIa was significantly higher in metastatic lesions than that of primary cancer tissues. Moreover, high level of FcγRIIIa was significantly associated with poor prognosis in PCa patients. We for the first time showed that FcγRIIIa was expressed in PCa cells from primary tumor tissues and metastatic lesions and PCa cell lines as well. FcγRIIIa expression was significantly higher in metastatic lesion compared to that of primary tumor tissues. We showed that *FCGR3A* gene amplifications and mRNA upregulation accounted for 9% of the metastatic PCa cases, in which 61% cases had AR gene amplifications and mRNA upregulation, 19% had *PIP5K1A* gene amplification and mRNA upregulation, and 37% cases had *PTEN* gene mutation, deletion, or mRNA downregulation. Furthermore, PCa patients with higher FcγRIIIa mRNA expression in their tumors had worse biochemical recurrence (BCR)‐free survival compared to those with lower FcγRIIIa expression. Our data suggest that FcγRIIIa expression is highly clinical relevant and may reflect its role in PCa development and progression.

In this study, we aimed to investigate whether FcγRIIIa may play an important role in growth and invasion of PCa at both AR‐dependent and AR‐independent fashions. We found that induced overexpression of FcγRIIIa in C4‐2 cells promoted cancer cell growth. Conversely, inhibition of FcγRIIIa via siRNA‐mediated knockdown reduced growth ability of C4‐2 cells. Similarly, induced overexpression of FcγRIIIa led to increased expression of AR and PIP5Kα, the key factors that promote PCa growth and invasion, while inhibition of FcγRIIIa led to decreased expression of AR and PIP5K1α.

One of the striking findings in this study is the identification of the underlying mechanism by which FcγRIIIa and AR interact with each other in PCa cells. We found that FcγRIIIa was capable of inducing AR target gene promoter activation as determined by using the ARE reporter luciferase activity assays. Furthermore, overexpression of AR led to significant increase in FcγRIIIa protein expression, while inhibition of AR by using enzalutamide decreased FcγRIIIa protein expression readily after 12 h of enzalutamide treatment of LNaP cells. In addition, we showed that AR and FcγRIIIa interacted with each other through formation of protein–protein complexes together with PIP5K1α. Our findings suggest that the observed effect of FcγRIIIa on AR may not be the consequence of FcγRIIIa‐induced cell proliferation in AR‐expressing PCa cells, but rather due to that FcγRIIIa is functionally associated with AR and PIP5K1α associated pathways via protein–protein interactions.

Interestingly, induced overexpression of FcγRIIIa in androgen‐independent PC‐3 cells that do not express functional AR also promoted proliferation and invasion of PC‐3 cells. Conversely, inhibition of FcγRIIIa using siRNA‐mediated knockdown led to significant decrease in growth and invasion of PC‐3 cells *in vitro* and PC‐3 tumors in xenograft mice. Although the underlying mechanisms by which FcγRIIIa promotes PCa growth and invasion at AR‐independent fashion remain obscure, our findings in *in vitro* and *in vivo* model systems provide strong evidence, suggesting that FcγRIIIa plays an important role in AR‐independent fashion. It has been reported that activation and expression of FcγRIIIa in immune cells are associated with the formation of immune complexes, and increased FcγRIIIa expression can lead to the subsequent activation of PI3K/AKT pathways in immune cells [1]. It is known that FcγRIIIa is activated by IgG immune complexes. Thus, the interaction between FcγRIIIa and IgG immune complexes is critical for FcγRIIIa internalization to enable the activation of FcγRIIIa downstream signaling events related to migration and survival of leukocytes [2]. It will be of great interests to investigate whether FcγRIIIa may utilize the IgG immune complexes from the PCa‐associated immune cells/tumor microenvironment to promote growth and progression of castration‐resistant PCa.

Our results further showed that PIP5K1α was functionally associated with FcγRIIIa, as inhibition of both PIP5K1α and FcγRIIIa resulted in greater inhibition of invasiveness of PCa cells as compared with inhibition of FcγRIIIa alone. Further, these key molecules organize and activate several signaling pathways, leading to tumor cell survival and invasion. We plan to further investigate the role of FcγRIII and the underlying mechanisms in PCa progression from androgen dependence to castration‐resistant state in the near future.

In this study, we applied xenograft models Targeted inhibition of FcγRIIIa via siRNA‐mediated knockdown or using inhibitory antibody suppressed growth of primary prostate tumors and reduced distant metastasis in xenograft mouse models. We further established novel metastatic xenograft mouse models to examine the effect of inhibition of FcγRIIIa activity on PCa metastasis. Further, our findings suggest that FcγRIIIa plays an important role in PCa progression and is a potential therapeutic target for the development of the new treatment strategies for advanced and metastatic PCa. Since elevated activity of FcγRIIIa can be inhibited using blockade antibody M3G8, we therefore examined the effect of M3G8 on PCa tumor growth and metastasis. Our data showed that M3G8 significantly suppressed tumor growth *in vitro* and in xenograft mouse models. M3G8 blocks both FcγRIIIa and FcγRIIIb, we found that M3G8 treatment led to an inhibition of FcγRIIIa and reduced expression of PIP5K1α/AKT, as determined by our immunoblot analysis by using antibody against FcγRIIIa, Further, the inhibitory effect of M3G8 treatment on PCa tumor growth is comparable to the effect of FcγRIIIa knockdown on PCa tumor growth.

Several previous studies have demonstrated that FcγRIIIa is a signal molecule that induces rapid and transient PIP5K1α membrane recruitment on NK cells to facilitate cytotoxic killing [43]. It is likely that PCa cells utilize FcγRIIIa to mimic immune cells and to evade cytotoxic cell‐mediated antitumor immunity.

## 5 Conclusions

Our results showed that treatment approach by optimizing activity to blocking antibody to FcγRIIIa is likely the good strategy to improve the therapeutic outcome by using antibody‐mediated destruction of malignant cells. Taken together, our findings suggest that FcγRIIIa may serve as a potential new target for the improvement of treatment of metastatic and castration‐resistant PCa.

## Conflict of interest

The authors declare no conflict of interest.

## Author contributions

RK, PFL, MS, RM, NØ, and JLP designed experiments. RK, PFL, MS, RM, TW, ASSK, AH, AA, SC, JS, AK, AS, and KEH performed experiments. RK, PFL, MS, RM, TW, ASSK, AH, AJ, SC, JS, AA, AS, NPM, DH, TG, BR, and JLP performed data analysis. RK, PFL, MS, TW, ASSK, NPM, DMH, SNW, DÖ, TG, NØ, and JLP contributed to major manuscript writing. All authors contributed to final editing and final approval of the manuscript.

### Peer review

The peer review history for this article is available at https://publons.com/publon/10.1002/1878‐0261.13166.

## Supporting information


**Fig. S1**. In the MSKCC/DFCI patient cohort consisting primary PCa (*n* = 1013 PCa cases) from the Prostate Oncogenenome Project dataset in cBioPortal databases, *FCGR3A* gene alterations were found in 3% of PCa cases, which was similar to *PIP5K1A* gene alterations accounted for 5% in this PCa cohort.
**Fig. S2**. Dot plots graph shows the correlations between AR and FcγRIIIa mRNA expression in log 2 by using the SU2C/PCF metastatic PCa cohort (*n* = 429). The *R* ^2^ vaule and *P* value are indicated.
**Fig. S3**. Dot plots graph shows the correlations between AR and FcγRIIIa mRNA expression in log 2 by using the TCGA PCa cohort (*n* = 333). The *R* ^2^ vaule and *P* value are indicated.
**Fig. S4**. Expression of FcγRIIIa in PC‐3 cells and U‐937 cells.
**Fig. S5**. FcγRIIIa protein expression in PC‐3 cells along with various types of PCa cell lines by using immunoblot analysis. U‐937 monocytes and PCa cell lines including C4‐2, VCaP, PC‐3 and PC‐3M cells were subjected to the immunoblotting analysis. Antibodies against FcγRIIIa and GAPDH were used.
**Fig. S6**. The effect of AR overexpression on FCGR3A mRNA expression. AR overexpression was induced in LNCaP cells by transfecting the cells with a vector carrying full‐length AR or a control vector. The semi‐quantitative RT‐PCR analysis using the primers specific for FCGR3A was performed.
**Fig. S7**. The effect of AR inhibition on FCGR3A mRNA expression. LNCaP cells were treated with enzalutamide for 6 h, 12 h and 24 h respectively. The effect of AR inhibition by enzalutamide on the expression of AR was measured by quantitative RT‐PCR.

## Data Availability

All data analyzed for this study are included in this published article and its supplemental information files. The data will be made available from the corresponding authors upon reasonable request.
