# Supplementary material for: FcγRIIIa receptor interacts with androgen receptor and PIP5K1α to promote growth and metastasis of prostate cancer
Source: Mol Oncol. 2022 Jan 23;16(13):2496–517. doi: 10.1002/1878-0261.13166 (PMC9251882; doi:10.1002/1878-0261.13166)
Supplement: Supplementary file 1 — Fig. S1. In the MSKCC/DFCI patient cohort consisting primary PCa (n = 1013 PCa cases) from the Prostate Oncogenenome Project dataset in cBioPortal databases, FCGR3A gene alterations were found in 3% of PCa cases, which was similar to PIP5K1A gene alterations accounted for 5% in this PCa cohort. Fig. S2. Dot plots graph shows the correlations between AR and FcγRIIIa mRNA expression in log 2 by using the SU2C/PCF metastatic PCa cohort (n = 429). The R 2 vaule and P value are indicated. Fig. S3. Dot plots graph shows the correlations between AR and FcγRIIIa mRNA expression in log 2 by using the TCGA PCa cohort (n = 333). The R 2 vaule and P value are indicated. Fig. S4. Expression of FcγRIIIa in PC‐3 cells and U‐937 cells. Fig. S5. FcγRIIIa protein expression in PC‐3 cells along with various types of PCa cell lines by using immunoblot analysis. U‐937 monocytes and PCa cell lines including C4‐2, VCaP, PC‐3 and PC‐3M cells were subjected to the immunoblotting analysis. Antibodies against FcγRIIIa and GAPDH were used. Fig. S6. The effect of AR overexpression on FCGR3A mRNA expression. AR overexpression was induced in LNCaP cells by transfecting the cells with a vector carrying full‐length AR or a control vector. The semi‐quantitative RT‐PCR analysis using the primers specific for FCGR3A was performed. Fig. S7. The effect of AR inhibition on FCGR3A mRNA expression. LNCaP cells were treated with enzalutamide for 6 h, 12 h and 24 h respectively. The effect of AR inhibition by enzalutamide on the expression of AR was measured by quantitative RT‐PCR. [file MOL2-16-2496-s001.docx]

**Supplemental information**


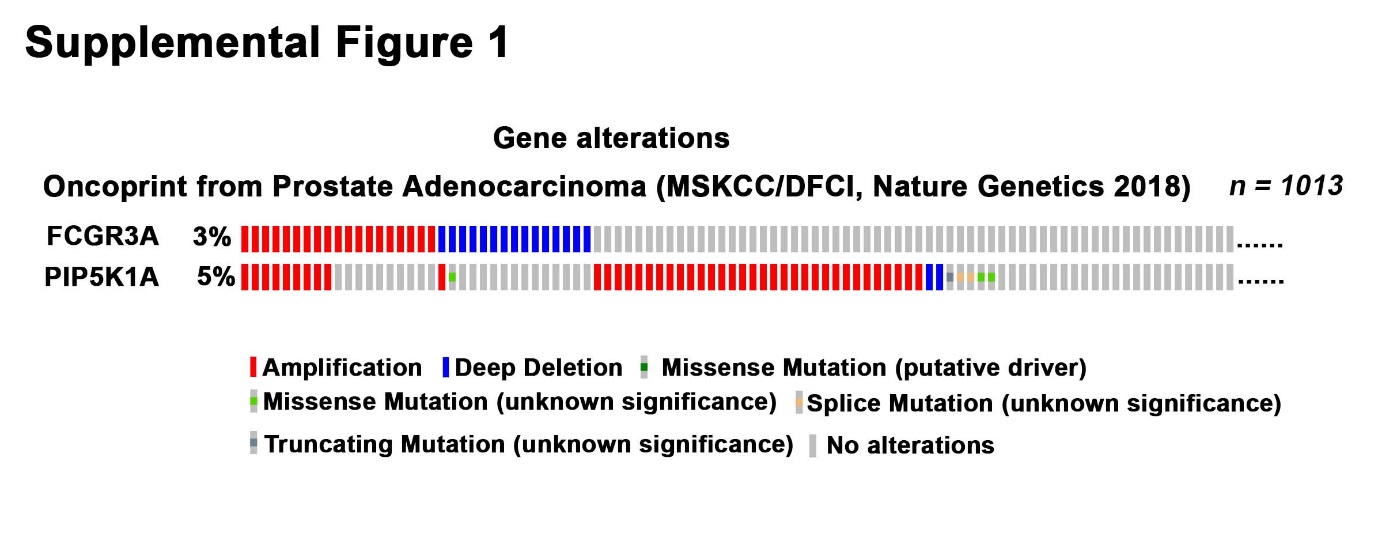


**Supplemental Figure 1.** In the MSKCC/DFCI patient cohort consisting primary PCa (n=1013 PCa cases) from the Prostate Oncogenenome Project dataset in cBioPortal databases, *FCGR3A* gene alterations were found in 3% of PCa cases, which was similar to *PIP5K1A* gene alterations accounted for 5% in this PCa cohort


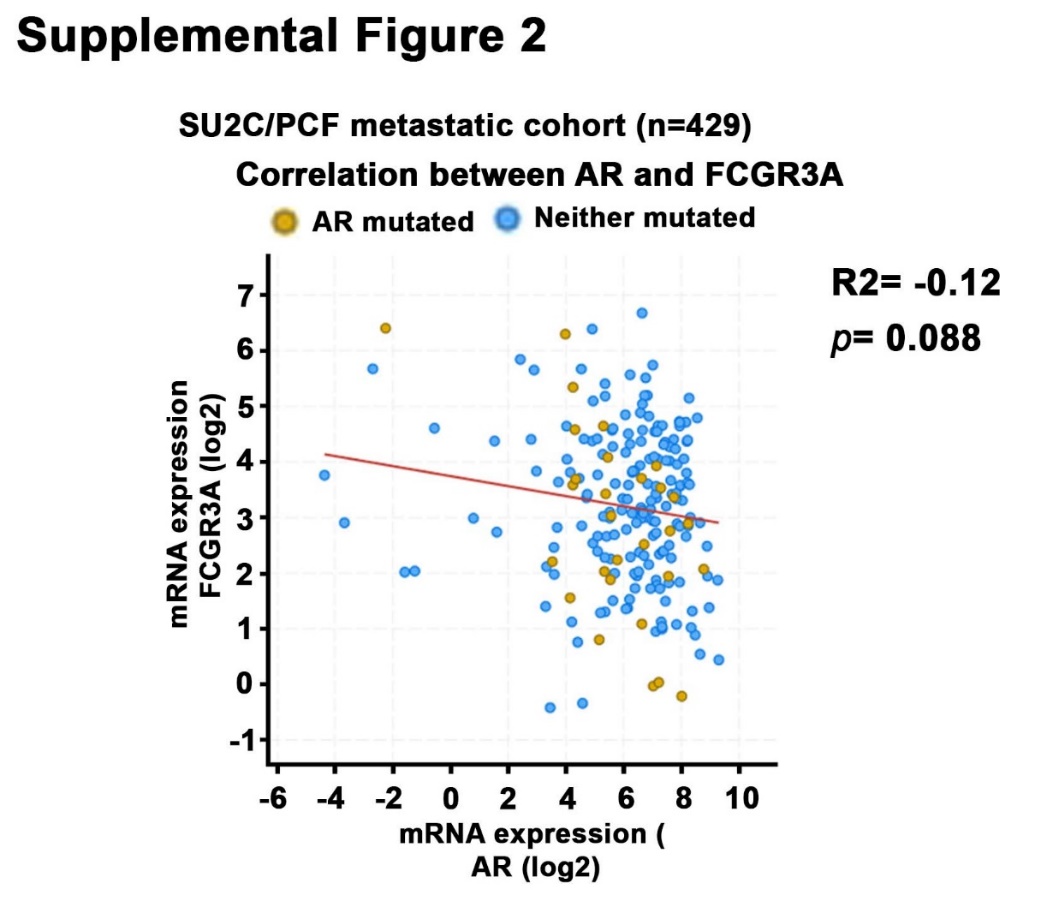


**Supplemental Figure 2.** Dot plots graph shows the correlations between AR and FcγRIIIa mRNA expression in log 2 by using the SU2C/PCF metastatic PCa cohort (n=429). The R2 vaule and p value are indicated.


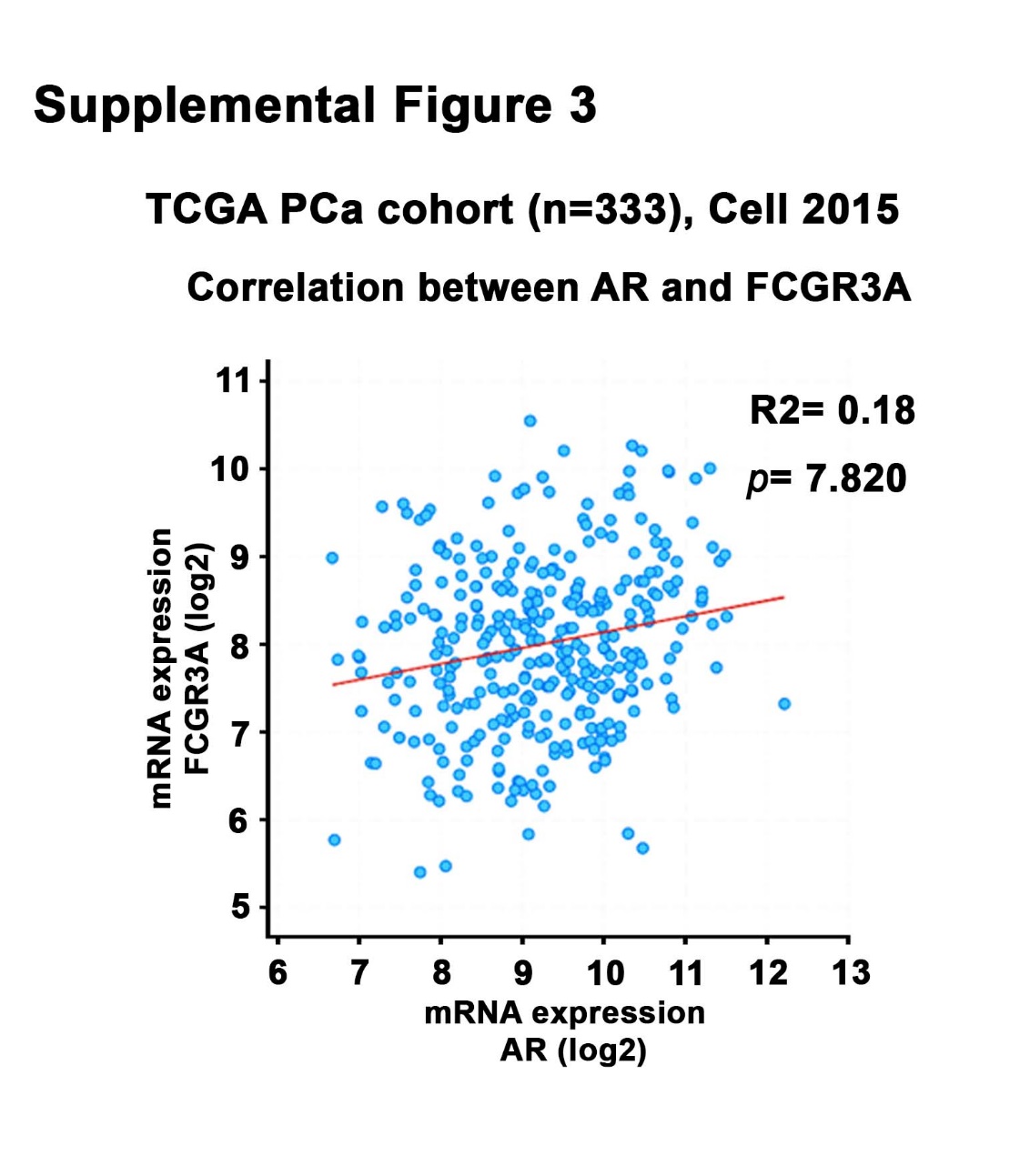


**Supplemental Figure 3.** Dot plots graph shows the correlations between AR and FcγRIIIa mRNA expression in log 2 by using the TCGA PCa cohort (n=333). The R2 vaule and p value are indicated.


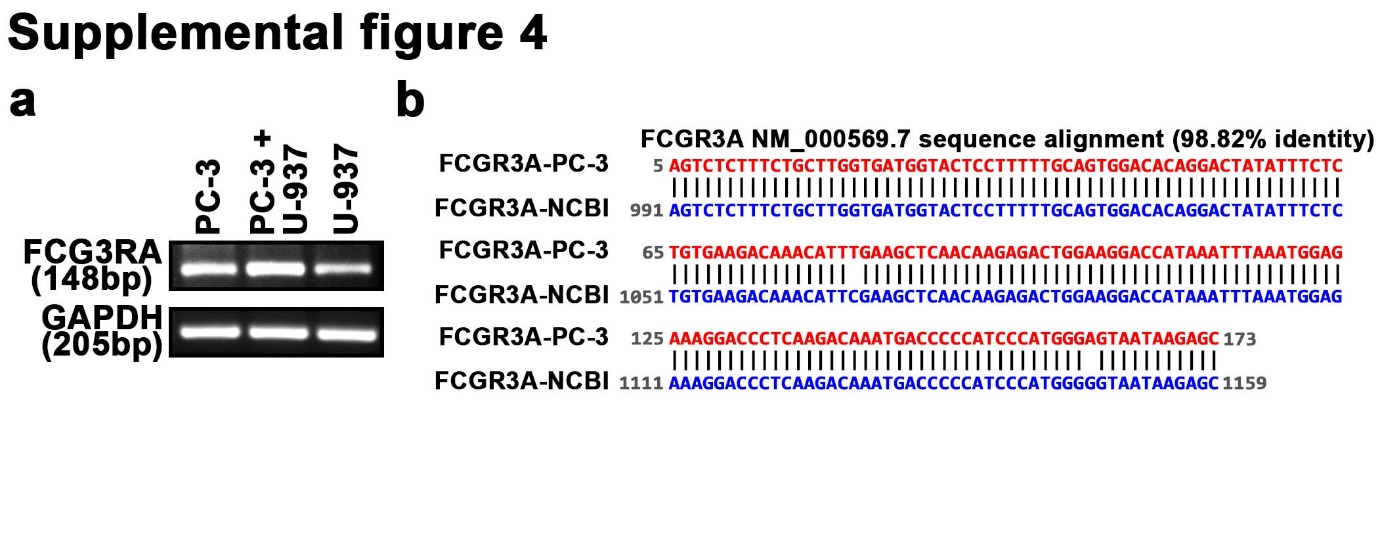


**Supplemental Figure 4:** Expression of FcγRIIIa in PC-3 cells and U-937 cells. (a). Semi-quantitative RT-PCR using the primers specific for *FCGR3A* was performed. The PCR-product of *FCGR3A* in mono-cultured PC-3 cells, U-937 cells and PC-3 after co-culturing with U-937 cells, the cells from each condition were collected after 48 hours in the culture. (b). The sequence alignment of the PCR-product of *FCGR3A* from PC-3 cells with the consensus sequence from NCBI is shown.


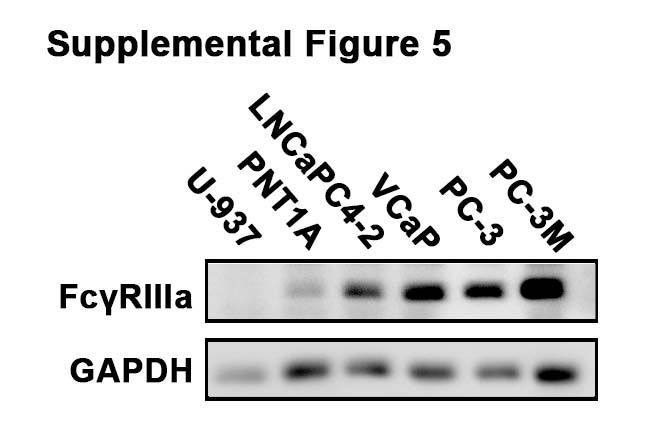


**Supplemental Figure 5.** FcγRIIIa protein expression in PC-3 cells along with various types of PCa cell lines by using immunoblot analysis. U-937 monocytes and PCa cell lines including C4-2, VCaP, PC-3 and PC-3M cells were subjected to the immunoblotting analysis. Antibodies against FcγRIIIa and GAPDH were used.


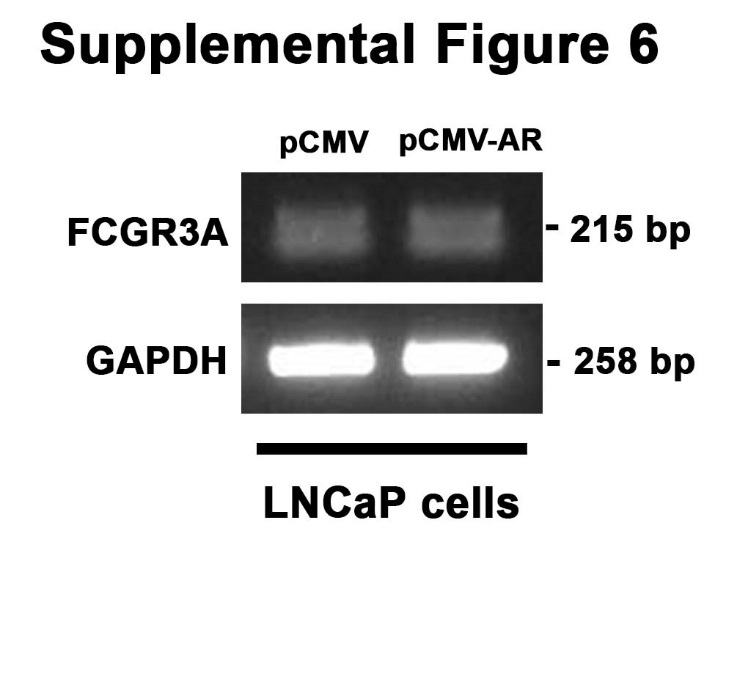


**Supplemental Figure 6**. The effect of AR overexpression on FCGR3A mRNA expression. AR overexpression was induced in LNCaP cells by transfecting the cells with a vector carrying full-length AR or a control vector. The semi-quantitative RT-PCR analysis using the primers specific for FCGR3A was performed.


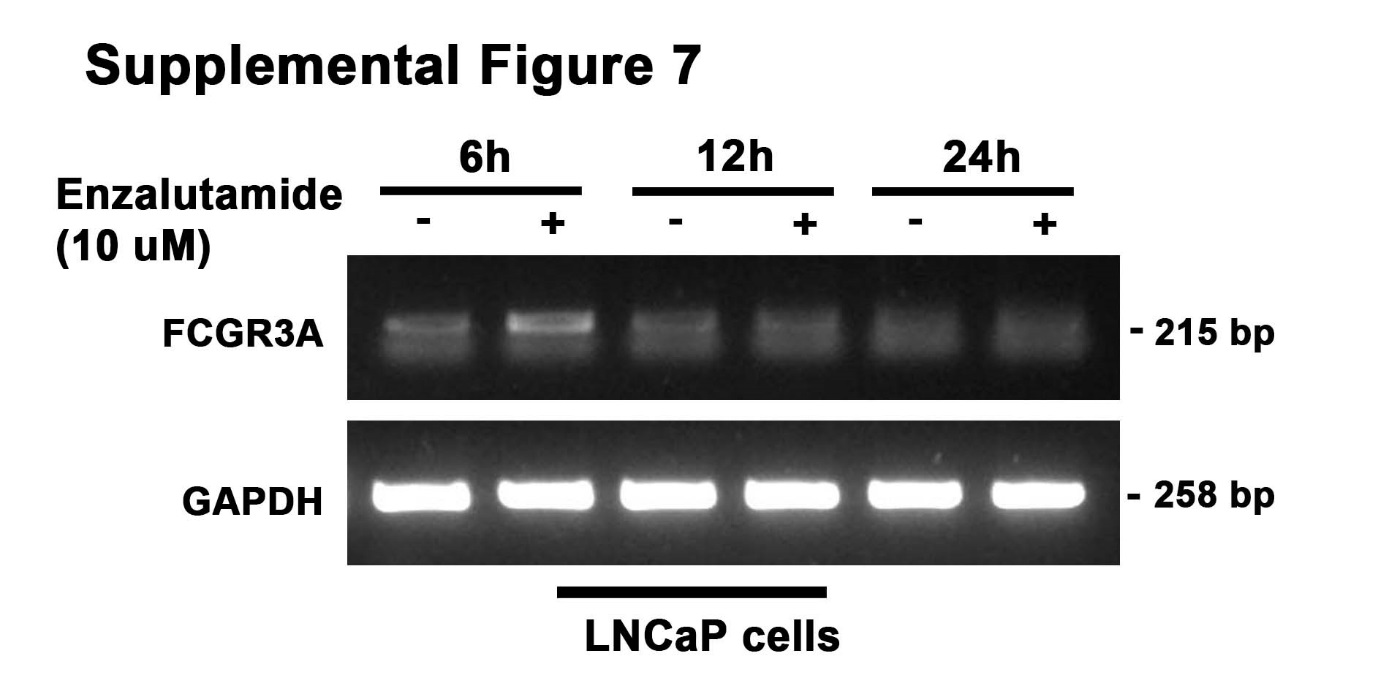


**Supplemental Figure 7.** The effect of AR inhibition on FCGR3A mRNA expression. LNCaP cells were treated with enzalutamide for 6 hours, 12 hours and 24 hours respectively. The effect of AR inhibition by enzalutamide on the expression of AR was measured by quantitative RT-PCR.
